# Polysaccharides for the Delivery of Antitumor Drugs

**DOI:** 10.3390/ma8052569

**Published:** 2015-05-13

**Authors:** Bianca Posocco, Eva Dreussi, Jacopo de Santa, Giuseppe Toffoli, Michela Abrami, Francesco Musiani, Mario Grassi, Rossella Farra, Federica Tonon, Gabriele Grassi, Barbara Dapas

**Affiliations:** 1 Centro di Riferimento Oncologico, Via Franco Gallini 2, I-33081 Aviano (PN), Italy; E-Mails: bposocco@cro.it (B.P.); edreussi@cro.it (E.D.); jdesanta.@cro.it (J.S.); gtoffoli@cro.it (G.T.); 2 Department of Engineering and Architecture, University of Trieste, Via Alfonso Valerio, 6/A, I-34127 Trieste, Italy; E-Mails: mario.grassi@di3.units.it (M.G.); rfarra@units.it (R.F.); effe.tonon@gmail.com (F.T.); 3 Department of Life Sciences, Cattinara University Hospital, Trieste University, Strada di Fiume 447, I-34149 Trieste, Italy; E-Mails: mikystars@hotmail.com (M.A.); bdapas@units.it (B.D.); 4 Laboratory of Bioinorganic Chemistry, Department of Pharmacy and Biotechnology, University of Bologna, I-40127 Bologna, Italy; E-Mail: Francesco.musiani@unibo.it

**Keywords:** polysaccharides, anticancer drugs, NABDs, delivery

## Abstract

Among the several delivery materials available so far, polysaccharides represent very attractive molecules as they can undergo a wide range of chemical modifications, are biocompatible, biodegradable, and have low immunogenic properties. Thus, polysaccharides can contribute to significantly overcome the limitation in the use of many types of drugs, including anti-cancer drugs. The use of conventional anti-cancer drugs is hampered by their high toxicity, mostly depending on the indiscriminate targeting of both cancer and normal cells. Additionally, for nucleic acid based drugs (NABDs), an emerging class of drugs with potential anti-cancer value, the practical use is problematic. This mostly depends on their fast degradation in biological fluids and the difficulties to cross cell membranes. Thus, for both classes of drugs, the development of optimal delivery materials is crucial. Here we discuss the possibility of using different kinds of polysaccharides, such as chitosan, hyaluronic acid, dextran, and pullulan, as smart drug delivery materials. We first describe the main features of polysaccharides, then a general overview about the aspects ruling drug release mechanisms and the pharmacokinetic are reported. Finally, notable examples of polysaccharide-based delivery of conventional anti-cancer drugs and NABDs are reported. Whereas additional research is required, the promising results obtained so far, fully justify further efforts, both in terms of economic support and investigations in the field of polysaccharides as drug delivery materials.

## 1. Introduction

Polysaccharides can be defined as polymeric carbohydrate structures composed of repeating monosaccharide units joined by glycosidic bonds [1]. These natural polymers are, by far, the most abundant renewable resource on the Earth, with an annual formation rate exceeding the world production rate of synthetic polymers by some orders of magnitude. Interestingly, naturally designed polysaccharides can carry out various different functions. For example, they can accomplish structural tasks, such as cellulose and chitin, storage functions, such as starch and glycogen, and gel structures (made up of mono- and copolymers), such as mucopolysaccharides (glycosaminoglycans), agar, and pectins [2].

Polysaccharides found and continue to find wide application in the drug delivery field [3,4,5,6,7,8] for various and important reasons: (1) they can be obtained in a well characterized and reproducible way from natural sources [9]; (2) they can undergo a wide range of chemical and enzymatic reactions to give origin to different materials [10]; (3) they are biocompatible, biodegradable and have low immunogenic properties [11]; (4) ionic polysaccharides can, partially, or even totally, substitute synthetic polymers in the design of stimuli responsive drug delivery systems since they show pH- and ion-sensitiveness [12]; (5) ionic polysaccharides are muco-adhesive [13]; (6) they can be prepared as conjugates or complexes with proteins, peptides, and other bio-macromolecules [14]; (7) they can easily form gels [14]; and (8) they can give rise to interpenetrated polymeric networks (IPN) and semi-IPN that can show physico-chemical properties which are remarkably different from those of the macromolecular constituents [15]. All these characteristics make polysaccharides excellent materials for the realization of “smart” delivery systems capable to release, at the appropriate time and site of action, entrapped drugs in response to specific physiological stimuli [15].

The aim of this review is to present relevant polysaccharides and to show some of their most significant applications in the drug delivery fields. More in detail, in Section 2 we will provide a general overview of polysaccharides structure, types, and delivery/absorption mechanisms; in Section 3 and Section 4 we report examples of the use of polysaccharides as delivery materials for clinically used anticancer drugs and experimental nucleic acid based drugs (NABDs), chosen as relevant examples among existing drugs.

## 2. Polysaccharides

### 2.1. Polysaccharide Structure

**Figure 1:**
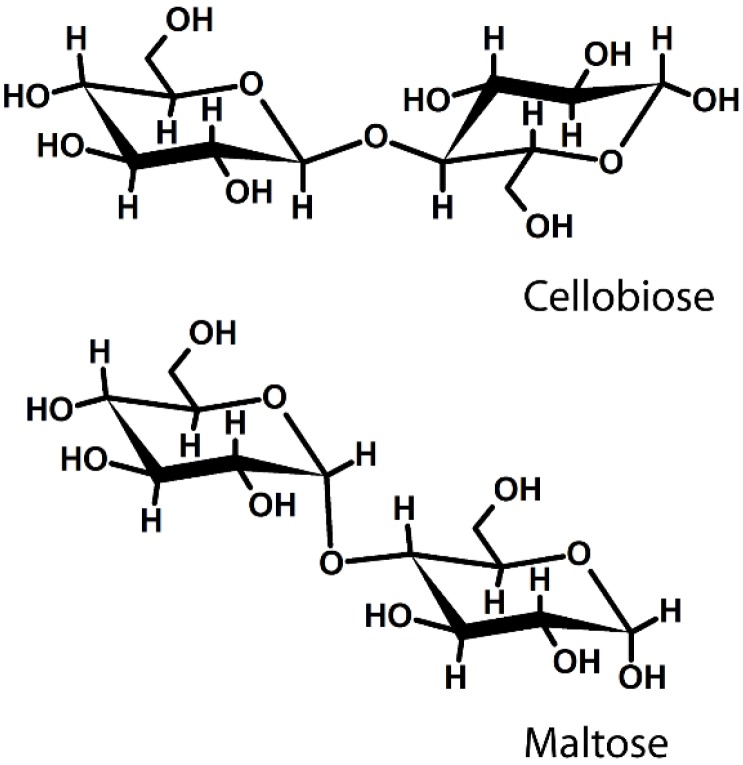
Glycosidic linkage between two different saccharide units. Schematic representation of cellobiose (**top**) and maltose (**bottom**). In the first case, the anomeric carbon (C2) is in the beta configuration (equatorial), while in the second case, it is in the alpha configuration (axial).

Polysaccharide classification basically relies on their “primary or covalent structure” that represents the sequence of monomeric units along the chain [2,10]. These units are, usually, characterized by a heterocyclic structure, where one atom is oxygen and the remaining are carbon atoms. The most common repeating units, which naturally occur in the polysaccharide chains, present a six-membered ring structure. Despite the carbon atom numbers, the monomeric units are linked by chemical bonds of covalent nature that connect the carbon atom in position 1 (*C*(1)) through an oxygen bridge to a carbon atom of the following unit via the so-called “glycosidic linkages” (Figure 1).

**Figure 2:**
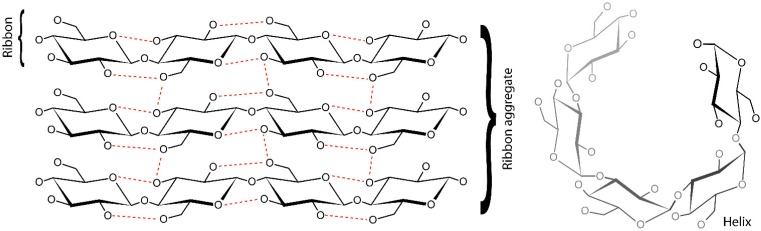
Schematic representation of ribbons and a ribbon aggregate (**left**), as found in cellulose, and of a helix (**right**), as found in starch. Hydrogen bonds are represented by dashed red lines.

As these covalent linkages are not completely flexible (they limit the monomers to a narrow range of relative orientations), an isolated polysaccharide chain can only adopt certain shapes (called “secondary structures”), which are dependent on their primary sequence. At a higher degree of organization, energetically favorable interactions between shaped chains may result in ordered specific structures known as “tertiary structures”. Finally, such compact entities may interact among themselves to give higher levels of organization, which are described as “quaternary structures”. Common examples of polysaccharide “secondary structures” (called conformations) are ribbons and helices. “Tertiary structures” are represented by the arrangement of multiple helices or aggregates of ribbons, as shown in Figure 2, in the case of cellulose and starch.

### 2.2. Polysaccharides Types

As shown in Table 1, polysaccharides can have animal, vegetal (algae, plants, seeds, and exudates), and microbial/fungi origins.

Therefore, they hold a wide range of different functions. Polysaccharides can act as energy storage materials, such as starch, glycogen, and polysaccharides of some plant seed (locust bean gum and guar gum). Polysaccharides can also contribute to the structural integrity and mechanical strength of plant tissues by forming a hydrated cross-linked three-dimensional network, as in the case of pectins in land plants and carrageenans, agar, and alginate in marine species. In the animal world, hyaluronate, chondroitin sulfate and other related glycosaminoglycans play a fundamental part in governing the solution properties of some physiological fluids and in the structure of the intercellular matrix. Polysaccharides may also work as protective substances, as in the case of exudate gums from plants, where they provide a preventive function by sealing off the injured parts of the plant against bacterial infections.

**Table 1:** Origin of Polysaccharides.

**Animal Polysaccharides**	
Chitin	
Chitosan [16,17,18,19,20,21,22,23,24,25,26,27,28]	
Glycosamminoglycans [29,30,31,32,33,34,35,36,37,38]	
**Vegetal Polysaccharides**	
**Land Vegetal**	**Marine Vegetal**
Cellulose [39,40,41]	Alginate (Phaeophyceae—brown seaweed)
Pectins	Agar (Rhodophyceae—red seaweed)
Galactomannans	Carragenans (Rhodophyceae—red seaweed)
Acacia gum	–
Starch	–
**Microorganisms/Fungi**	
Alginate (*Pseudomonas aeruginosa* and *Azotobacter vinelandii*) [42,43,44]	
Dextran (*Leuconostoc spp.* and *Lactobacillus spp.*) [45,46,47,48,49,50]	
Gellan (*Pseudomonas elodea*)	
Pullulan (*Aureobasidium pullulans*) [51,52,53]	
Scleroglucan (*Sclerotium glucanicum*) *Xanthomonas campestris*	
Xanthan (*Xanthomonas campestris*)	

#### 2.2.1. Animal Polysaccharides

Chitin, a high molecular weight polysaccharide, is a fundamental skeletal material in crustaceans, insects, and spiders that contributes to the structure of the exoskeleton, the lining of the gut, the tendons, the wing coverings, and the internal skeleton. The primary structure of chitin is closely related to that of cellulose (linear polymer of 1,4-linked β-d-glucopyranose), except for the replacement of the –OH group at each *C*(2) position by an acetamido group –NHCOCH_3_. Chitosan is the name given to fully or partially deacetylated chitin.

Glycosaminoglycans (GAGs) are polysaccharides that contain amino sugars and are constituents of the extracellular space of many mammalian tissues. With the exception of hyaluronic acid, they are covalently linked to proteins in the natural state, and in this form, they are known as proteoglycans. Hyaluronic acid is a basic component of the extracellular matrix of connective tissue and it is present in large amounts in the vitreous humour of the eye, the synovial fluid of the joints, and in soft tissues, such as umbilical cord and rooster comb. In addition, hyaluronic acid has many peculiar chemical–physical and biological properties, which enable it to regulate and control the physiological functions of several tissues *in vivo*. In virtue of its extraordinary water-retaining capability, in aqueous solutions hyaluronic acid shows a high excluded volume since it behaves as an expanded random coil occupying large hydrodynamic volume. This is the reason why it is generally affirmed that it controls and regulates the trafficking of fluids and macromolecules within the interstitial space of tissues [54].

#### 2.2.2. Vegetal Polysaccharides

Cellulose, a high molecular weight (≈7 × 10^5^–3.2 × 10^6^ [10]) linear polymer of 1,4-linked β-d-glucopyranose, can be defined as the backbone of the vegetal kingdom as it is one of the most abundant and important component of land-growing plants.

Pectins represent a group of polysaccharides that occur as structural materials in all land-growing plants. They belong to the interrupted chain sequences family, being characterized by linear 1,4-linked α-d-galactopyranosyluronic acid sequences separated by α-l-rhamnopyranosyl residues. Galactomannans represent a class of polysaccharides that can be found in plant seeds such as carob or locust bean, guar, tara, and tamarind. These polysaccharides, or gums as they are often called, are derived from the endosperm of various leguminosae plant seeds, where they function as reserve materials utilized during germination. Most of these carbohydrate polymers share basic structural similarities consisting of linear 1,4-linked chains of β-d-mannopyranosyl residues to which single α-d-galactopyranosyl side chains are linked at position 6. Depending on the seed source, galactomannans can differ in their molecular weight, molecular weight distribution and galactose to mannose composition ratio (*G*/*M*). Moreover, at a given *G*/*M* ratio, they can also differ in terms of the side chain distribution along the mannan backbone. Gum Arabic, or acacia gum, is the most famous natural exudate, produced by more than 900 species of the Acacia trees. Natural Arabic gum is a mixture of potassium, magnesium and calcium salt of an acidic polysaccharide composed of six different carbohydrate units: d-galactopyranose, d-arabinopyranose, l-arabinofuranose, l-rhamnopyranose, d-glucopyranosyluronic acid and 4-*O*-methyl-d-glucopyranosyluronic acid.

A common function of polysaccharides that preferentially adopt hollow helix conformations is to act as energy reserves in biological systems. For these biopolymers, the conformations are relevant to their biological functions as they allow the macromolecules to be piled together and accumulated as concentrated deposits. These deposits can be organized in such a way that osmotic transport phenomena within the tissues are not affected and, yet, the molecules within them are easily accessible to be mobilized and employed when necessary. A typical example of energy reserve polysaccharide is represented by starch that, in nature, occurs as minute granules (2–100 μm particle size) in the roots, seeds, and stems of numerous types of plants including corn, wheat, rice, millet, barley, and potatoes. Starch is made up of a linear polymer, called amylose (1,4-linked α-d-glucopyranose units), and a branched polymer called amylopectin (linear backbone of 1,4-linked α-d-glucopyranose monomers bearing clusters of 1,6-α-linked glucopyranosyl branches with average length of 20–30 residues). Amylose constitutes only the 25% by weight of the total starch.

Polysaccharides can be also found in marine seaweeds or algae. Their task is similar to that of cellulose in land plants, with the difference that seaweeds need a more flexible structure to support the varying stresses due to currents and waves. The many varieties of seaweeds are usually subdivided on the basis of their predominant color pigments. Accordingly, Cyanophyceae (blue-green), Xanthophyceae (yellow-green), Chlorophyceae (green), Rhodophyceae (red), and Phaeophyceae (brown) can be mentioned. Among the many polysaccharides present in seaweeds and algae (see Table 1), from the drug delivery point of view, the most important are alginates and agar. The term alginate refers to a family of unbranched polysaccharides isolated from brown seaweeds [55]. They are composed of 1-4-linked β-d-mannuronic acid (*M*) and alpha-l-guluronic acid (*G*) arranged in a block-wise pattern with homopolymeric regions of *M*-blocks and *G*-blocks residues interspersed by regions of alternating structures (*MG*-blocks). The polysaccharide, known since 1881, has largely been used in biotechnological and industrial applications for its gel forming properties, which were described in detail around the 1970s by Grant [56] and Smidsrød [57].

The general term agar refers to a complex mixture of polysaccharide components which can be extracted from certain species of red seaweeds. Agar is composed of two major fractions: agarose, a neutral polysaccharide, and agaropectin, a charged, sulfated carbohydrate polymer. The percentage of agarose normally ranges from 50% to 90% of the total agar. The linear agarose backbone is based on a disaccharide repeating unit of the *AB* type, consisting of alternating 1,3-linked β-d-galactose (*A*) and 1,4-linked 3,6-anhydro-α-L-galactose (*B*). Depending on the algal sources, 6-*O*-methyl-d-galactose may also be present in variable amounts (1%–20%). Interestingly, agarose can give origin to thermo-reversible hydrogels.

Carrageenans are sulfated linear polysaccharides which can be extracted as matrix components of red seaweeds. Their chemical structure is based on a disaccharide repeating unit of the *AB* type so that they can be written generally as (*AB*)*_n_*, where *A* is derived from a 1,3-linked β-d-galactose unit and *B* is usually, but not always, present as 1,4-linked 3,6-anhydro-β-d-galactose. Carrageenans sulfation takes place at position 2 and 4 in 1,3-linked galactose residues, whereas the 1,4-linked units occur as 2-sulfate, 6-sulfate, 2,6-disulfate and 3,6-anhydride-2-sulfate. No sulfation at *C*(3) apparently occurs.

#### 2.2.3. Microorganisms/Fungi Polysaccharides

Microorganisms, such as bacteria and fungi, produce three distinct types of polysaccharides: (1) extracellular or exo-cellular polysaccharides, which can be found in the form of a discrete capsule that envelops the microbial cell and that is part of the cell wall itself (capsular polysaccharides), or as an amorphous mass secreted into the surrounding medium; (2) structural polysaccharides; and (3) intracellular storage polysaccharides.

The bacterial alginate is exo-cellularly produced (partially acetylated) by the bacteria *Pseudomonas aeruginosa* and *Azotobacter vinelandii* and its physical and chemical properties are very similar to their algal counterparts. *O*-acetylation invariably seems to be a characteristic feature in bacterial alginates that distinguishes them from the seaweed alginates.

Dextrans constitute a family of microbial polysaccharides produced by *Leuconostoc spp.* and *Lactobacillus spp.* Dextran is almost exclusively composed of 1,6-linked α-d-glucopyranose units with varying proportions of other bond types, such as α-1,3, α-1,2 and α-1,6 branch linkages. Gellan gum is produced by the bacterium *Pseudomonas elodea* and is composed of d-glucose, d-glucuronic acid and l-rhamnose in a molar ratio of 2:1:1. The primary structure of gellan is based on the tetra-saccharide repeating unit. Both *O*-acetyl and *O*-l-glyceryl substituents are present along the main chain, the former at the *C*(6) position and the latter at the *C*(2) position of the 3-linked glucose unit. The exo-cellular Pullulan, produced by the yeast-like fungus *Aureobasidium pullulans*, is a linear polymer composed of glucose residues polymerized into repeating malto-triose units. The malto-triose units are linked via α-1,6 bonds; within each unit the glucose residues are linked α-1,4. Nevertheless, some 1,6-α-d-maltotetraose repeating units and 1,3-linked glucose residues have also been found as components of pullulan primary structure. The molecular weight of this polysaccharide can vary in the range 1.5 × 10^3^–4 × 10^6^ depending on the culture conditions and strain of microorganisms used.

Scleroglucan, a neutral capsular polysaccharide, is exo-cellularly secreted by certain imperfect fungi, particularly of the genus Sclerotium. The primary structure of scleroglucan from *Sclerotium glucanicum* has been characterized as a linear chain of β-1,3-linked d-glucose units with single d-glucose side chains linked β-1,6 to every third unit of the main chain. Scleroglucan molecular weight varies in the range 3.2 × 10^5^–1 × 10^6^. Xanthan gum is an anionic polymer secreted exo-cellularly by the bacterium *Xanthomonas campestris*. Its primary structure is based on a linear backbone of 1,4-linked β-d-glucose. At the *C*(3) position of every alternate glucose residue there is a charged tri-saccharide side chain containing a glucuronic acid residue between two mannose units. The terminal β-d-mannose is linked β-1,4 to the glucuronic acid, which, in turn, is linked α-1,2 to the α-d-mannose. In approximately one half of the terminal mannose residues, a pyruvic acid moiety is joined by a ketal linkage to the *O*(4) and *O*(6) positions. Acetyl groups are present as substituents at the *O*(6) position of the nonterminal d-mannose.

### 2.3. Polysaccharide Gels

**Figure 3:**
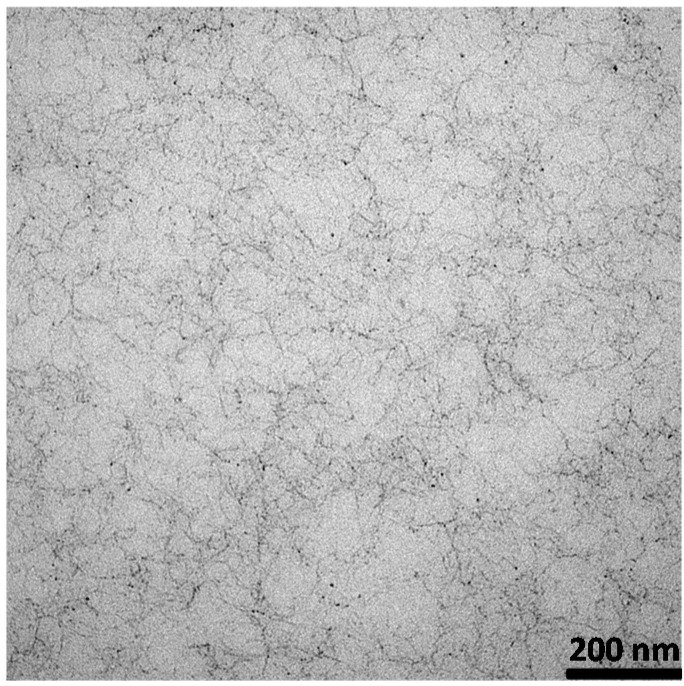
TEM image referring to a hydrogel obtained via the crosslinking of an alginate water solution (alginate mass fraction 2%) by means of a *Ca*^++^ solution (5 g/L). Alginate chains are represented by the dark wires while water occupies the gray zones. Details about the hydrogel treatment for the TEM analysis are reported in [60].

Undoubtedly, among the different interesting properties of polysaccharides, the possibility of forming gel structures is, from the drug delivery point of view, very interesting. Indeed, gels, defined as coherent systems made up of a three-dimensional polymeric network trapping a continuous liquid phase (water or physiological media in the case of hydrogels) [10] (see Figure 3), can efficiently and reliably control the drug release kinetics of embedded drugs [58]. The presence of inter-chains linkages, called crosslinks, prevents the solubilization of the network of the polymeric chains in the fluid phase. Interestingly, despite the very large amount of liquid phase that can be hosted inside the polymeric network (the polymeric network volume fraction in the gel can be lower than 1%), gels show mechanical properties in between those of solids and liquids [10], thus, mimicking living tissues very well [59].

The chemical–physical properties of the polymeric network chains and the huge amount of absorbed water, make polysaccharide gels attractive candidates also for the release of proteins, peptides, vaccines and NABDs that can be embodied into the three-dimensional polymeric network meshes. The use of polysaccharide as delivery systems is also strengthened by the fact that hydrogel realization implies the use of safe procedures that do not imply proteins/NABDs denaturation. Thus, controlled release systems, based on polysaccharides hydrogels, can be a reliable tool to ensure the therapeutic proteins/peptides/NABDs concentration in tissues or in the blood for a long time [14].

**Figure 4:**
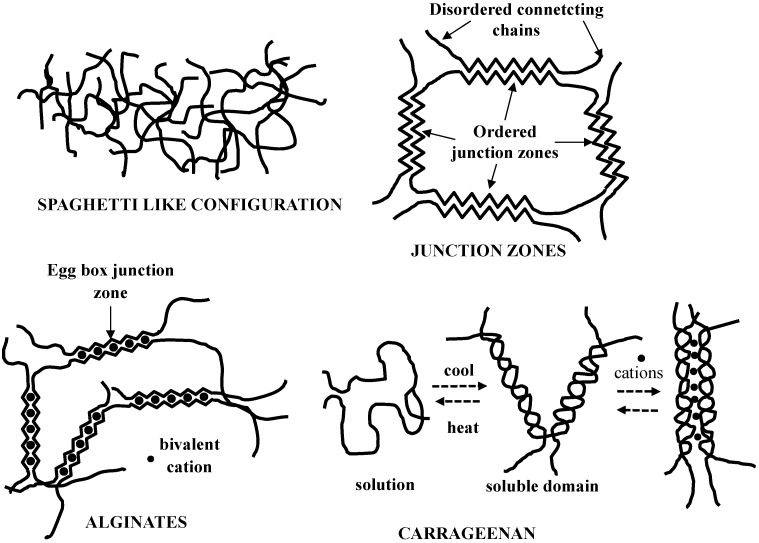
Structures occurring in physically cross-linked polysaccharides gels.

Based on the crosslinked nature, (polysaccharides) hydrogels can be defined as chemical or physical. In the first case, crosslinks between different chains are strong, permanent, and punctual as they are due to covalent bonds. Conversely, in the second case, crosslinks are due to either polymer chains topological entanglements (spaghetti-like configuration, Figure 4) or physical interactions (this being typical of glucans and xanthan), such as H-bonds, ionic, Coulombic, van der Waals, dipole–dipole, and hydrophobic interactions.

As a consequence, crosslinks are not punctual but they form junction zones where the regular coupling of chain portions belonging to different polymeric chains takes place. It is interesting to observe that the long chain segments departing from the ordered junction zones can form, with other chains, additional junction zones to form a polymeric three-dimensional network. As, in general, the physical interactions occurring in junction zones are not so strong, these junctions are often transient. Thus, they give origin to a statistical network characterized by a constant average crosslink density (moles of crosslinks per gel unit volume) and a time dependent distribution of crosslinks. These means that, due to Brownian motion of chains and segment of chains, network meshes continuously appear and disappear, being the average mesh number and dimension constant [10,58]. Figure 4 shows some typical situations occurring in the formation of polysaccharide gels.

Basically, drug delivery from hydrogels as those formed by polysaccharides, is ruled by physical processes, physicochemical processes, and system parameters [61]. In particular: (1) physical processes can include matrix swelling and erosion; (2) physicochemical processes can include matrix erosion, drug dissolution (recrystallization), drug transport (by diffusion and convection), drug interaction with hydrogel polymeric chains; and (3) system parameters can be represented by initial drug distribution and concentration inside the hydrogel, hydrogel geometry (cylindrical, spherical, *etc*.), and hydrogels size distribution (in the case of an ensemble of hydrogel particles). Of course, among all the above mentioned phenomena, those strongly affected by the hydrogel network topology are swelling, erosion, and drug diffusion. On the contrary, network physicochemical characteristics can play an important role for what concerns the drug interaction with the polymeric chains. In general, if the network topology highly affects the drug release kinetics from hydrogels ranging in the milli- or micro-range, polymer–drug interaction becomes the most important aspect for polymeric nano-delivery systems.

### 2.4. Polysaccharide Micro and Nanoparticles

Whereas polysaccharides can be used in the form of gel to deliver therapeutic drugs, they can be also arranged in the micro-nanoparticle shape. This last use has been limited in the past due to the harsh conditions necessary to prepare the particles, a fact that may compromise drug stability and loading efficiencies [62,63]. A recently developed approach to overcome this limitation is represented by the generation of super-hydrophobic surfaces [62]. This method is based on the preparation of a water solution containing the monomer (or polymer) and the drug at the desired concentration. Then, the aqueous solution is put on a super-hydrophobic surface constituted, for example, by surface modified copper, aluminum, or polystyrene substrates. Due to the high hydrophobicity of these surfaces, the aqueous solution assumes an almost perfect spherical shape whose diameter essentially depends on the solution volume deposited on the surface. The subsequent exposure to the polymerisation/crosslinking process leads to the formation of spherical particles [63]. Since the process takes place at the solid–air interface, no migration of the drug occurs, and, thus, 100% loading yield is possible [62]. Relying on this approach, Lima and co-workers [63] were able to realize spherical hydrogel particles, made up by interpenetrated dextran-methacrylated and poly(*N*-isopropylacrylamide) polymers, for the release of insulin and bovine serum albumin. Interestingly, they proved that the release kinetics could be tuned acting on particles composition and release environment temperature.

Whatever the technique used to prepare the drug loaded particles, the *in vivo* fate of the particles depends on some common steps reassumed by the ADME processes, *i.e.*, Absorption, Distribution, Metabolism and Elimination [64]. In the case of particulate delivery systems, such as those based on polysaccharide particles, cell membrane crossing (absorption) represent a critical step. In particular, the net superficial charge of the particles plays a key role. Whereas anionic complexes usually show good solubility and, thus, stability in the physiological environment, they cannot efficiently cross cell membranes. This is due to the electrostatic repulsion occurring with the negative electric charge of the cell membranes. Conversely, cationic complexes can efficiently bind to cell membrane due to the strong electrostatic interactions. Unfortunately, however, if the interaction is too strong, it can cause membrane disruption and consequent cell death. In addition, the presence of negatively charged blood proteins can induce the formation of complex-protein aggregates that are no longer soluble and precipitate. Whereas neutral complexes are not affected by the above mentioned problems, in the physiological environment they tend to associate each other resulting in a limited solubility. Thus the charge of the particles has to be carefully selected. Ideally the particle charge should guarantee a good stability in the physiological environment and should be able to allow an efficient interaction with the cell membrane without inducing significant damages.

Although very important, the surface properties are not the only variables affecting the *in vivo* particle/drug fate. In particular, the particle dimension and shape can affect the transport through biological barriers (e.g., microvascular walls, interstitial space, and cell membranes) [65]. It is well known that, in general, small nanoparticles (≤100 nm) are efficiently internalized by cells, while bigger particles are not [66]. However, nanoparticles suffer for the lack of the so called “margination effect” which consists in the movement of particles in flow toward the walls of a channel (blood vessel) [67]. Essentially, nanoparticles move inside the vessel (together with red blood cells) with a uniform radial distribution and limited near-wall accumulation. On the contrary micro-particles tend to preferentially accumulate next to the vessel walls, in a particle size and shape-dependent manner [65,67]. This localization facilitates micro-particle extravasation, thus favoring drug delivery to the surrounding tissue. In contrast, nanoparticles, which have a limited near-wall accumulation, have more difficulties to extravasate and thus to deliver the drug to the tissues. The margination phenomenon is particularly relevant in vessels nourishing tumor tissues due to their leaky nature (enhanced permeability and retention effect) which favors extravasation [67]. Thus, the extravasation of particles localized near the vessel wall (microparticles) is facilitated; in contrast, the extravasation of particles localized far from the vessel wall (nanoparticles) is disfavored.

## 3. Polysaccharide-Based Delivery Systems for Anti-Cancer Drugs

Most, if not all, the concepts expressed in Section 2.3 and Section 2.4, with regard to the variables ruling drug delivery from polysaccharides, hold true for any drug delivery approach. This implies that the works reported in the next sections are the result of the complex interplay among all the aspects ruling drug release either from polysaccharide gels or particles. To make the text easily understandable, these aspects are generally omitted in the description of the experimental works; the focus is in contrast put on some other features peculiar of the specific polysaccharide-based delivery system.

The presented works have been subdivided into two parts: the first (Section 3.1) describing examples of the release of clinically relevant anticancer drugs, and the second (Section 3.2) describing examples of the release of nucleic acid based drug.

### 3.1. Polysaccharides for the Delivery of Clinically Relevant Anti-Cancer Drugs

In addition to the drug delivery aspects reported in Section 2.3 and Section 2.4, other features specific to the delivery of clinically relevant anti-cancer drugs, have to be highlighted. We believe this is important to better understanding the rationale of the polysaccharide-based delivery examples described. For this purpose, in Section 3.1.1 and Section 3.1.2 are reported some important aspects of drug delivery together with the main features of commonly used drugs, respectively. These two sections are then followed by the description of different polysaccharide-based delivery systems (Section 3.1.3, Section 3.1.4, Section 3.1.5, Section 3.1.6, Section 3.1.7 and Section 3.1.8).

#### 3.1.1. Critical Aspects of Systemic Drug Administration

The critical bottleneck of the administration of conventional cancer therapeutic molecules is represented by their high toxicity [68,69], mostly dependent on the indiscriminate distribution between diseased and healthy cells. Toxicity may also depend on the presence, in the pharmaceutical preparations, of organic solvents/detergents necessary to improve the poor solubility in water of many of these drugs [70]. Therefore, the development of targeted drug delivery systems with switchable (generally pH-dependent) drug release profiles has become a relevant issue in chemotherapy [71].

**Figure 5:**
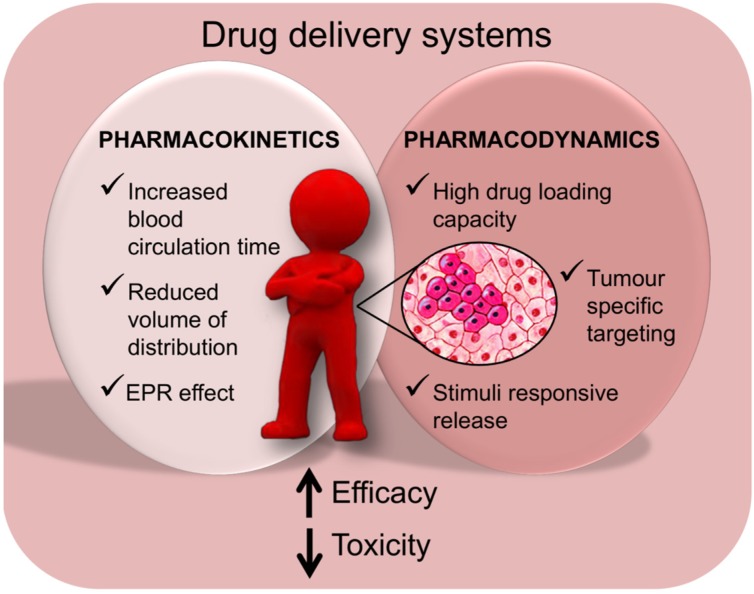
Advantages of chemotherapy agent conjugation with macromolecules.

The conjugation of chemotherapy agents to macromolecules may offer several advantages (Figure 5).

Macromolecules can ameliorate drugs’ pharmacokinetics (PK) and pharmacodynamics (PD) by different phenomena. Regarding PD, macromolecules have high drug loading capacity and, in addition, can be equipped with targeting molecules to increase drug specificity. In some cases, stimuli-responsive delivery has been studied to improve drug release in cancer tissues. In this regard, for example, pH-sensitive drug delivery systems take advantage of the different pH in normal (pH~7.4) *vs.* cancer (pH < 6) tissue [72]. With regard to PK, macromolecules can increase blood circulation time, reduce the volume of distribution and prolong the distribution/elimination phases [73]. It is also noteworthy that, in general, polymer–drug conjugates are not able to cross the endothelium surrounding healthy vessels. This mostly depends on the bigger size of polymer–drug conjugates compared to endothelium fenestration. In contrast, in pathological conditions like cancer, local vessels are more permeable due to the increased size of endothelium fenestration. Through these gaps, the delivery systems can extravasate and reach the tumor where they tend to reside due to the scarce lymphatic drainage. Collectively these phenomena go under the name of enhanced permeability and retention (EPR) effect [74]. The use of materials able to release the drug slowly, may further improve drug residence at the tumor site, possibly ameliorating drug efficacy and reducing side effects.

The use of macromolecule-based delivery systems also allows facing more complex clinical problems. For instance, it is possible to design multidrug delivery systems to improve, on one hand, the treatment efficacy and, on the other hand, the compliance of the patients. Another interesting option is related to the possibility to design so called “theragnostic” agents, that render possible the simultaneous cancer diagnosis and therapy. This approach typically combines the use of a therapeutic agent together with a targeting/marker agent, as below detailed.

Based on the above considerations, chemotherapeutic drugs have been encapsulated, conjugated or linked to different carriers [75]. Among these, biopolymers have been frequently used due to their biocompatibility, natural occurrence, often targeting ability, relatively inexpensiveness and the possibility of derivatization with different chemical groups [76].

#### 3.1.2. Anticancer Drugs

Thus far, a great deal of effort has been spent to design polysaccharide-based systems to deliver anti-cancer drugs such as docetaxel (DTX), paclitaxel (PTX), cisplatin (CDDP), 5-fluorouracil (5-FU), and doxorubicin (DOX).

DTX and PTX are mitotic inhibitors belonging to the taxane family. These molecules promote the assembly of the free tubulin and stabilize the microtubules, thus, particularly impacting proliferating cells. According to FDA guidelines, DTX is indicated for the treatment of various solid tumors such as breast cancer, non-small cell lung cancer, hormone refractory prostate cancer, gastric adenocarcinoma, squamous cell carcinoma of head and neck. Among the numerous side effects reported, toxic deaths, hepatotoxicity, neutropenia, and hypersensitivity reactions can be mentioned. Another important limitation of DTX relates to its scarce water solubility that, together with the reported side effects, have lead numerous groups to try to ameliorate drug formulation and administration. As DTX, PTX is widely used in clinic for the treatment of breast cancer, ovarian cancer, and AIDS-related Kaposi sarcoma. The aforementioned side effects depend not only on the drugs, but also on the additives used to improve their poor solubility, like the surfactant polysorbate [77,78].

Another class of drugs commonly used in cancer treatment are DNA damaging agents. Among these, CDDP is employed for the therapy of many malignancies including metastatic testicular tumors, metastatic ovarian and advanced bladder cancer. Its administration is associated with many side effects due to its accumulation in the liver, kidney, and prostate.

5-FU is an antimetabolite that acts by interfering with the DNA and RNA synthesis. It is prescribed for the management of different malignancies such as carcinoma of the colon, rectum, breast, stomach, and pancreas. As for other chemotherapeutics, severe hematological and gastrointestinal toxicities and even toxic deaths are reported.

DOX, belonging to the class of anthracyclines, has mainly three different mechanisms of action. First, it binds to nucleic acids, intercalating the planar anthracycline nucleus with the DNA double helix; second, it interacts with topoisomerase II, an enzyme involved in DNA replication; third, it binds to the cellular membrane, affecting a variety of cellular functions. This drug is indicated for different cancers, such as bone sarcomas, Hodgkin’s disease, ovary-, breast- and lung-cancer [79]. Among the side effects, it can be stressed in particular the cardiotoxicity and the possibility to develop acute myelogenous leukemia or myelodysplastic syndrome.

To improve drug effectiveness but especially to reduce side effects, different delivery options have been developed. In the next sections, we will concentrate on those based on the use of polysaccharides (Table 2). Most of the examples reported deal with the above-presented anticancer drugs; however, also other chemotherapeutics are reported and in these cases, their characteristics are briefly indicated.

**Table 2:** Polysaccharide-based delivery of chemotherapy drugs.

	System	Structure	Drug	Disease	*In vitro* Model	*In vivo* Model	Ref.
**Chitosan**							
1	LMWC-PTX	LMWC	PTX	Melanoma, NSCLC	–	Mouse	[16]
2	LMWC-DTX	LMWC	DTX	NSCLC, GBM	NCI-H358, U87MG	Mouse	[17]
3	PTX-Cy5.5-CS	NPs	PTX	SCC	SCC7	Mouse	[18]
4	Cy5-CS	NPs	DOX	Sarcoma	HT1080	–	[19]
5	HGCS-Ce6	NPs	Ce6	SCC, adenocarcinoma	SCC-7	Mouse	[20]
	GCS-Ce6				HT-29		
6	FLT-CS	MPs	FLT	–	–	–	[21]
7	CS/GP	Hydrogel	CDDP	Oral cancer	–	–	[22]
	CS/GP/GE						
**Hyaluronic Acid**							
8	MSP-HA-DOX	Nano-conjugate	DOX	Breast cancer	MDA-MB-231	–	[29]
9	HA-DOX	Nano-conjugate	DOX	Breast cancer	MDA-MB-468NL	Rat	[30]
10	HA-CDDP	Nano-conjugate	CDDP	Lung cancer	A549	Rat	[31]
11	HA-CA-PTX	Nano-conjugate	PTX	Head and neck cancer	SCC7, NIH3T3	Mouse	[32]
12	HACD-AuNPs	Gold nanocluster	several	Breast cancer	MCF7, NIH3T3	–	[33]
**Dextran**							
13	CMD-g-β-CD/(PAD-g-AD&AD-DOX))_4_	Microsystem	DOX	–	HeLa	–	[45]
14	DEX-PDP	Nanosystem	DOX, CPT11	Breast cancer	MCF7, DLD1	–	[46]
				Colon cancer			
**Pullulan**							
15	P-PTX	NPs	PTX	Colon cancer	HCT116	Mouse	[51]
**Cellulose**							
16	HPC-DNR	Conjugate	DNR	–	HeLa	Mouse	[39]
17	CMC-PABA, CMC-PAH	Nano-conjugate	Curcumin and derivates	Colon cancer	HT29, CRL1790	–	[40]
18	HPC-PAA-CdSe	Nanogel	TMZ	Melanoma	B16F10	–	[41]
**Other**							
19	MR-5-FU	NPs	5-FU	Breast cancer	MCF7	–	[80]
20	FA-AG-MTX	NPs	MTX	–	AA8	–	[81]

#### 3.1.3. Chitosan Based Delivery

Chitosan (CS) has been considered as delivery material because of its unique physicochemical and biological characteristics. The primary hydroxyl and amine groups located on the backbone of CS allow chemical modification to control its physical properties. The chemical modifications allow, for example, overcoming the low solubility at physiological pH and the poor buffering capacity of CS in its native form. Additionally, when a hydrophobic moiety is conjugated to a CS molecule, the resulting amphiphile may form self-assembled nanoparticles (NPs) that can encapsulate a variety of hydrophobic drugs and promote proper delivery [71].

Several examples of drug delivery systems based on CS have been reported in literature. Low molecular weight CS (LMWC) (MW < 10 KDa) conjugated with PTX (LMWC-PTX) through a succinate linker, was synthesized as a NP carrier for the oral delivery of PTX [16]. The antitumor efficacy of LMWC-PTX was evaluated in murine melanoma cells and in xenografted human non-small cell lung carcinomas (NSCLC) after oral administration. The strong antitumor activity was attributable to the improved water solubility, prolonged retention in the gastrointestinal tract and the ability to bypass the efflux pump of the gastrointestinal, cells as well as the metabolism in the intestine and liver. Moreover, LMWC could open quickly and reversibly the tight junctions between human epithelial colorectal adenocarcinoma cells (Caco-2), a useful characteristic that can improve intestine absorption [71,82].

Following an analogous approach, LMWC was also conjugated with DTX always through a succinate linker [17]. Cytotoxicity of LMWC-DTX conjugate was evaluated by MTT assay in human NSCLC-NCI-H358 and brain glioblastoma (U87MG) cell lines, showing similar effectiveness compared to the naked DTX. However, LMWC-DTX conjugate showed markedly enhanced (>200 times) water solubility compared to DTX alone. Moreover, following oral administration of LMWC-DTX in normal female BALB/c mice, blood half-life was increased by ~15-fold in comparison to the intravenously injected DTX. The *in vivo* antitumor efficacy was evaluated in nude mice bearing NCI-H358 and U87MG cells, respectively. The orally administered LMWC-DTX conjugate (10 mg DTX equivalent/kg) showed comparable antitumor efficacy to the same dose of the intravenously injected DTX for both NCI-H358 and U87MG models, but revealed much lower sub-acute toxicity as evaluated considering body weight loss and hematological toxicity in treated animals.

Another use of CS involves the generation of theragnostic NPs. In this case NPs carry a diagnostic and a therapeutic molecule enabling diagnosis, therapy, and monitoring of therapeutic response at the same time [83]. The possibility of exerting “Therapeutic Drug Monitoring” allows in principle the individual adjustment of drug dosing. This implies that it may be possible to optimize the therapy for any specific individual reaching a “personalized treatment” which is expected to be more effective and less toxic than available therapeutic protocols. CS-based theragnostic NPs, produced as spherical structures with approximately 260 nm in diameter, were loaded with PTX and linked with the fluorescent dye Cy5.5 for imaging [18]. NPs were then administered intravenously to mouse xenografted with head and neck squamous cell carcinoma cell line (SCC7). The high stability in serum, deformability and rapid uptake by tumor cells, allowed the NPs to localize to the tumor mass with minor uptake by normal tissues. Fluorescence imaging, used to directly monitoring tumor size, revealed that NPs were approximately two times more effective in tumor size reduction compared to naked PTX. The authors claimed that the superior tumor specificity of NPs was responsible for an increased drug accumulation in the tumor tissue, eventually resulting in a more potent antitumor effect. It would be interesting to see whether these NPs may exert the same targeting properties also with different tumor types and/or a different experimental set up. Despite this, the advantage of using CS NPs remains obvious compared to the administration of naked PTX.

More complex CS-based theragnostic NPs were generated with the possibility to monitor the drug release *via* Förster resonance energy transfer (FRET) [83]. A pH-responsive DOX-loaded NPs made of N-palmitoyl CS bearing a Cy5 fluorescent moiety (Cy5-CSNPs) were prepared [19]. The proper spatial positioning of DOX and Cy5 allowed FRET to occur. After endocytosis in the fibrosarcoma cell line HT1080, it was possible to observe that DOX fluorescence in the cytosol was barely seen when NPs were present in the slightly acidic early endosomes. However, after NPs moving to more acidic organelle (late endosomes/lysosomes), an improved release of DOX into the cytosol was detected. Consequently, a progressive accumulation into the cell nuclei occurred, paralleled by a significant DOX induced cytotoxicity. Notably, due to its mechanisms of action, DOX can exert its effect only reaching the nuclear compartment.

Photodynamic therapy (PDT) is a clinical treatment that employs photo-triggered chemical drugs as photosensitizers. To improve therapeutic efficacy and reduce the side effects in normal tissue, various nanoscale photosensitizer delivery systems have been developed [83]. As photosensitizers can generate both fluorescence and singlet oxygen upon laser irradiation, they can be used for both optical imaging and PDT at the same time [84]. In this regard, the hydrophobic photosensitizer chlorine e6 (Ce6) was loaded onto the hydrophobically-modified glycol CS NPs (average diameters of 300 to 350 nm) either by physical loading (HGCS-Ce6) or chemical conjugation (GCS-Ce6) [20]. Both HGCS-Ce6 and GCS-Ce6A displayed similar efficacy *in vitro* in the generation of singlet oxygen and a rapid cellular uptake in cultured SCC7. Compared to GCS-Ce6, HGC-Ce6 showed a burst of drug release *in vitro*, whereby 65% of physically loaded drugs were rapidly released from the particles within 6.5 h. However, following intravenous injection, HGCS-Ce6 did not accumulate efficiently in HT-29 human colon adenocarcinoma cells implanted in mice. In contrast, GCS-Ce6 had a prolonged circulation time and efficiently accumulated in the tumor, resulting in an excellent therapeutic effect. These findings suggest that the combined delivery of the two NP types may allow a fast delivery (HGC-Ce6) followed by slower release (GCS-Ce6), useful to maintain the pharmacological effects. The poor targeting ability of HGCS-Ce6 might be circumvented by adding targeting moieties on the NP themselves and/or including NPs in micro/macro delivery systems with targeting ability.

CS has been also utilized in form of microparticles (MPs) to deliver drugs. In the study of Elgindy *et al*. [21] CS-MPs (particle size range of 0.63–1.13 mm) were undertaken for the oral prolonged release of flutamide (FLT-CS-MPs). FLT is a nonsteroidal antiandrogenic agent recommended for prostate cancer monotherapy. FLT and its more potent active metabolite 2-hydroxyflutamide, competitively block dihydrotestosterone binding at androgen receptors, forming inactive complexes that cannot translocate into the cell nucleus. Formation of inactive receptors inhibits androgen-dependent DNA and protein synthesis, resulting in the growth arrest of tumor cell. Since FLT has low bioavailability after oral formulations due to low water solubility [85], Elgindy *et al*. [21] developed a water soluble formulation for a prolonged release. Simple ionic gelation and emulsification-ionic gelation techniques were used to prepare FLT-CS-MPs. Different formulations of FLT-CS-MPs were prepared and their ability to release the drug was tested *in vitro* at low pH to simulate the gastric environment. In general, a fast initial drug release at pH 1.2 was observed for the first two hours followed by a prolonged drug release for the remaining 10 h of the experiment. Notably, depending on the FLT-CS-MPs formulation, variants on this release behavior were possible. Although promising, this study is very preliminary and, thus, further studies in more complex experimental models are necessary to fully clarifying its efficacy.

In addition to the use in the form of NPs/MPs, CS has been also used as hydrogel for drug delivery. In this regard, there has been a great deal of interest in the use of hydrogels as chemotherapeutic drug delivery systems especially for oral administration. This is mainly due to the fact that the polymeric chains of the hydrogel can closely interact with the saliva glycoproteins, causing a muco-adhesion phenomenon [86]. For example, Moura *et al*. [22] investigated *in vitro* CDDP release from thermo-sensitive CS hydrogels cross-linked with glycerol phosphate disodium salt (CS/GP) (narrow pore size distribution centered around a mode of about 60 µm) and CS hydrogels that were ionic/covalently co-cross-linked with genipin (CS/GP/GE) (broad pore size distributions with larger pores which modes are around 200 µm). Both hydrogels could be produced *in situ* under physiologic conditions. Notably, the two types of hydrogels behaved differently as drug delivery systems. The amount of drug released was of about 20% for CS/GP and about 60%–70% for the CS/GP/GE hydrogel during the experimental time. Despite these differences, from the qualitative point of view the release profiles were similar for both hydrogel types being characterized by an initial burst, which reached a plateau following about 2–3 h. These systems are very attractive for the treatment of oral cancers because of the muco-adhesive property of the hydrogel, which can guarantee a prolonged permanence of the drug in the mouth. Moreover, the release kinetic can provide a first burst of the drug, useful for an immediate antitumor effect; then the slow release step is appropriate to maintain the therapeutic effects.

#### 3.1.4. Hyaluronic Acid Based Delivery

In mammalian organisms, native Hyaluronic acid (HA) represents one of the main constituents of the extracellular matrix (ECM). Moreover, HA can modulate cellular fate by receptor-mediated uptake. For example, HA can bind cluster determinant 44 (CD44), receptor for hyaluronate-mediated motility (RHAMM), HA receptor for endocytosis (HARE) and lymphatic vessel endothelial hyaluronan receptor-1 (LYVE-1), in all cases inducing specific cellular consequences. The above properties together with the intrinsic hydrophilicity, biocompatibility, biodegradability and non-immunogenicity, make of HA an excellent candidate for biomedical needs.

DOX delivery by HA has been addressed in various works. Mesoporous silica (MPS) is a small nanomaterial known for its ability to host a large amount of drug and releasing it following changes in pH, temperature and by competitive binding [87,88]. The group of Chen [29] had chemically functionalized MPS surface with two chemicals (1-ethyl-3-(3-dimethylaminopropyl)carbodiimide) EDC and 3-(2-aminoethylamino) propyltrimethoxysilane NHS. The functionalization allows a homogeneous coating of MPS with HA, which results in high stability in physiological solution. Additionally, the HA coating of MPS allows the interaction with the receptor CD44, which is frequently over-expressed in tumors [89,90] and permits the complex internalization. Once in the cell, HA is digested by HA-specific enzymes leading to drug release into the cytosol from uncoated MPS. Based on this principle, Chen *et al*. [29] have found that MPS-HA loaded with DOX (MPS-HA-DOX) can decrease the viability of the human breast cancer cell line (MDA-MB-231) more effectively (15%) than DOX alone. Additionally, when administered to the non-tumor cell line NIH3T3, MPS-HA-DOX displayed 20% reduced unspecific toxicity compared to DOX alone. The authors explained this last feature with the reduced [91] expression of CD44 on NIH3T3, a fact which somehow protected the cells from particle binding and, thus, from DOX action.

Another smart approach to deliver DOX by HA is exploiting pH sensitive modifications. An example of this strategy has been developed by Cai’s group [30]. Linking adipic acid hydrazide (ADH) to HA, the authors created a DOX (HA-DOX) conjugate. In this system the hydrazone bond (between HA and DOX) is hydrolyzed faster at pH 5 than pH 7.4, thus allowing the release of DOX in an acidic environment, such as that found in tumors and in internalization vesicles. The PK and toxicology features of the conjugate were studied in an MDA-MB-468NL (human breast adenocarcinoma cell line) orthotopic xenograft rat model. Compared to free DOX, HA-DOX, administered by subcutaneous injection, showed a reduced pick in plasma concentration (about 1/4). From the toxicology perspective, free DOX but not HA-DOX treatment induced renal and cardiac dysfunction. Moreover, HA-DOX conjugates reduced tumor growth more efficiently than naked DOX, with an increased animal survival rate of 50% over a time of 24 weeks. Even though the system has been tested only in one *in vivo* model, the results suggest its possible use in HA over-expressing tumors, a property which however needs to be further proven. Additionally, to test the CD44 specificity of the system, it would have been interesting to verify the effectiveness in CD44 non-over-expressing tumor cells.

The delivery of CDDP by HA based particles has been proposed by Xie *et al*. [31]. The authors tested a mono- and di-conjugate of CDDP and HA (HA-CDDP) in *in vitro* (A549 cell line, human lung adenocarcinoma epithelial cell line) and *in vivo* models (normal and xenografted rats) of lung cancer. The idea was to develop a local CDDP delivery to minimize the side effects that occur following CDDP intra veniously (*i.v*.) administration. Thus, the authors used the lung instillation route for HA-CDDP and compared the side effects with the classical *i.v*. administration of CDDP. Following lung instillation, HA-CDDP displayed better PK and reached higher concentrations in the lungs compared to *i.v*. administered naked CDDP. In particular, HA-CDDP reached 5.7- and 1.2-fold higher concentration than naked *i.v*. CDDP, 24 and 96 h after instillation, respectively. Notably, drug distribution to other tissues, such as brain, kidneys and liver was significantly more evident for naked *i.v*. CDDP compared to lung-instilled HA-CDDP. The more targeted localization of HA-CDDP resulted in reduced neuro- and nephron-toxic effects of HA-CDDP compared to CDDP. Whereas the investigation about the side effects are encouraging, future studies are required to examine the HA-CDDP therapeutic efficacy.

A curious and recent application of HA-coupled PTX has been undertaken by Thomas *et al*. [32]. The authors, by linking 5β cholanic acid (CA) to HA, produced micelle able to encapsulate and solubilize PTX in a ratio of 10:1 *w*/*w* (HA-CA-PTX). The study has been carried out *in vitro* in the cancer cell line SCC7 and in *in vivo* models (xenografted mice). The SCC7 cell line (CD44 over-expressing) and NIH3T3 (CD44 low-expressing) cells as control, were preliminary tested for the uptake using particles loaded with fluorescent dyes (Flamma™-552) but not PTX. In SCC7 cells, the uptake was superior for the dye containing HA-CA particles compared to naked dye; notably, the uptake was significantly superior in the CD44 over-expressing SCC7 cells compared to the CD44 low-expressing NIH3T3 cells, thus proving the targeting ability. With regard to the antitumor effect, HA-CA-PTX at various concentrations (0.01–100 µg/mL range), resulted to be from 10% to 40% more effective than naked PTX in SSC7. Bio distribution experiments *in vivo*, carried out using Flamma™-774 labeled particles administered intravenously, demonstrated that HA-CA particles accumulate in the liver from where they are cleared one day after administration; in contrast, the tumor localization was maintained up to three days. The harvested organs confirmed the particle accumulation in the tumor and in liver and evidenced some accumulation in the kidney. The *in vivo* efficacy tests conducted with 5 mg/kg of PTX in HA-CA micelles intravenously injected, showed a significant inhibition of tumor growth compared to naked PTX. Notably, whereas the maximum effect of naked PTX was observed at day 4 after administration, that of HA-CA-PTX occurred at day 8. This most likely reflects the different release kinetic, which may contribute to explain the improved therapeutic effect of HA-CA-PTX.

As a last example of HA mediated drug delivery, we mention the work of Li *et al*. [33]. This very promising, though not fully tested study, is based on the use of polysaccharide-gold nano-cluster. The system is constituted by a supramolecular carrier of gold NPs (AuNPs) bearing adamantine moieties and β-cyclodextrin-modified HA (HA-CD). The strong affinity between the β-CD cavity and the adamantane moieties allows the system to be stable [92]. Additionally, the system permits drug release in mild acidic environments. Besides these features, the system has other favorable properties: good biocompatibility [93,94], convenient synthesis and facile size control [95], robust stability under most *in vivo* conditions [96], tunable surface features and dense loading functionalities for specific cell targeting [97,98]. A further advantage of this carrier is represented by the possibility to be loaded with different drugs. In this regard, the authors demonstrated satisfactory loading abilities for both hydrophobic and hydrophilic drugs. A preliminary application of HACD-AuNP coupled with DOX in MCF7 breast cancer cells (over-expressing CD44), showed a significant reduction in cell viability, comparable to that observed with naked DOX. Notably, in NIH3T3 (CD44 low expressing), no significant cytotoxic effects were observed, suggesting that the CD44 targeting is the key determinant for HACD-AuNP/DOX effects. To confirm the targeting ability, it would have been interesting to test whether MCF7 pretreatment by HA receptor binding molecules could have prevented HACD-AuNP/DOX toxic effects. Despite this, the work is potentially interesting and constitutes the basis for further *in vivo* tests.

#### 3.1.5. Dextran Based Delivery

Dextran is considered a convenient delivery material due to its hydrophilic character, biocompatibility, biodegradability and easy chemical derivatization.

A notable example of dextran-mediated delivery has been reported by the group of Luo [45]. The authors developed a process to produce microcapsules fabricated via host-guest interaction between polyaldehyde dextran-graft-adamantane and carboxymethyl dextran-graft-β-cyclodextrin. This kind of interaction has strong stability and it is pH responsive, due to the presence of acidic-sensitive hydrazone bonds in polyaldehyde dextran-graft-adamantane. Adamantane was used as DOX linking site giving rise to a complex structure named carboxymethyl dextran-graft-β-cyclodextran/polyaldenhyde dextran-graft-adamantane and adamantine-modified DOX (CMD-g-β-CD/(PAD-g-AD and AD-DOX))_4_. The presence of pH responsive bonds was introduced to confer the ability to release the drug in the acidic cancer tissues. The pH responsiveness was tested first *in vitro*, where it was proved the particle stability and the capacity to release the drug in relation to acidification. In particular, at pH 5.5 about 80% of the loaded drug was released, while at physiological pH only 18% of the drug was delivered. The cytotoxic potential of the DOX loaded microcapsules was then tested in cultured HeLa cells (human cervical cancer cell line), at different pHs (7.4 and 5.5). At pH 7.4 only a small amount of DOX was absorbed and thus no important effect on cell viability was observed. Under acidic conditions, about 50% of loaded drug was released with a dramatic reduction in cell viability. The pH responsive property together with the excellent biocompatibility of the unloaded microcapsules, make this particle type very promising. Future tests in *in vivo* model are necessary to confirm the obtained results and to determine the PK and PD profiles.

Another interesting and innovative strategy has been proposed by Pramod *et al*. [46], who reported a dextran-based method able to deliver two different drugs from the same particle. Dextran nano-vesicles were generated with a dextran backbone bound to a hydrophobic pentadecyl phenol (DEX-PDP) group via an aliphatic ester linkage. As this bond can be hydrolyzed only by the lysosomial enzymes esterases, outside the cell environment it has a high stability. The vesicles can be loaded with hydrophilic molecules in the core and hydrophobic ones in the external layer. The amphiphilic nature allowed the concomitant loading with the water-soluble DOX and the hydrophobic irinotecan (CPT11), an inhibitor of topoisomerase I, of which cytotoxicity is related to double-strand DNA damage. According to FDA, CPT11 is recommended for the treatment of metastatic colorectal cancer. In the study of Pramod *et al*. [46], three different vesicle types were prepared: the first loaded with DOX, the second with CPT11 and the third one with both of them. Cytotoxicity and drug release were tested in MCF7 and DLD1 (human colon cancer) cell lines. In both cancer cell lines, the combined administration of DOX and CPT11 in a 4:1 ratio had the best effect in terms of cytotoxicity, killing 80% and 60% of MCF7 and DLD1 cells, respectively. Notably, the activity of the particles loaded with the two drugs was always superior to that of the same particles loaded with the two drugs separately. Tests in more complex *in vivo* models are now necessary to prove the effectiveness of the vesicles. Additionally, it may be interesting to test the effectiveness of vesicles loaded with different combinations of drugs.

#### 3.1.6. Pullulan Based Delivery

Pullulan (P) represents another potential interesting polysaccharide due to its biocompatibility, water solubility, lack of immunogenicity, and to the presence of multiple hydroxyl groups that can be functionalized. Notably, P can target the hepatic cells due to the ability to interact with the asialo-glycoprotein receptor present on hepatocytes. A notable example of P use is represented by the work of Lee *et al*. [51], where PTX-incorporated NPs have been synthesized using P acetate. NPs were spherical with a size from 80 nm for empty NPs and up to more than 100 nm for those loaded with PTX. *In vitro* it was demonstrated that PTX was released with an initial burst that lasted up to day two, followed by a reduced release over one week. In RAW264.7 macrophage cells, NPs alone were nontoxic and when loaded by PTX, they exerted a significant antitumor activity in the HCT116 human colon carcinoma cells. However, NPs-PTX were less effective than naked PTX, probably due to the slowed release kinetic. This features, which was detrimental in cultured cells was, on the contrary, the probable reason for the success *in vivo* in HCT116 xenografted in athymic nude BALB/c mice. Following *i.v.* administration and starting from ten days after drug administration, NPs-PTX demonstrated a superior activity than naked PTX in reducing tumor growth. Notably, compared to naked PTX, NPs-PTX induced a reduced body weight loss, suggesting a reduced systemic toxicity. The authors proposed that, in addition to the slowed release kinetic, the success of the increased effectiveness and reduced side effect of NPs-PTX was also dependent on the EPR effect. Whereas being fully acceptable, this hypothesis could have been verified by studying the distribution into the tumor tissue of NPs-PTX compared to naked PTX.

#### 3.1.7. Cellulose Based Delivery

Cellulose, due to the biocompatibility and anti-microbial action, is one of the most promising materials for bio-applications. Although it is well known that cellulose is insoluble in many solvents and water, it can be chemically modified for improving its solubility. There are several commercial soluble derivatives of cellulose, such as hydroxypropyl methylcellulose (HPC), a non-ionic, thermoresponsive and biodegradable compound [99]. Metaxa and coworkers [39] used HPC conjugated to daunorubicin (DNR), a DOX analogue, via a glutathione-sensitive linker (*N*,*N*'-(disulfanediylbis(ethane-2,1-diyl))bis(2-methylacrylamide) (DSBMA). In the presence of glutathione, the disulfide bridges of the polymeric layer are transformed into thiol groups, resulting in the slow release of DNR. As glutathione is highly concentrated in tumor cells [100], the system is in principle able to have a cancer cell specific effect. This feature may be further improved by the covalent modification of HPC with a folic acid receptor (FR) targeting moiety. Being that FR scarcely expressed in normal cells and over-expressed in a range of cancer cells, the system has an additional cancer cell targeting ability. The HPC-DNR particles were able to significantly reduce the vitality of the cancer cell line HeLa, over-expressing FR. In contrast, negligible effects were observed in the HEK293 (human embryonic kidney cell line), which express FR at low levels. Over a period of three days, HPC-DNR displayed reduced activity compared to naked DNR, probably due to the slow release kinetic. This hypothesis is supported by the observation that HPC-DNR could localize into HeLa cells. In particular, two hours after administration, the amount of HPC-DNR was higher compared to naked DNR, and negligible amounts were seen in the FR negative NIH3T3 cells. *In vivo* (mouse model), HPC-DNR displayed rapid clearance from circulation following intravenous administration and an uptake by the mononuclear phagocyte system. In this work, no evidence of the effects of glutathione on DNR release from HPC-DNR were presented; additionally, no evidence on the *in vivo* activity and specificity have been provided. Despite these aspects, the possibility to bind FR and release drug in high glutathione concentration, make the system interesting for further investigation.

Another cellulose type, carboxymethylcellulose (CMC), can be used for drug delivery because of its hydrophilic nature. Plyduang *et al*. [40] explored this material to deliver tetrahydrocurcumin (THC). THC is the active metabolite of curcumin, a naturally occurring antioxidant, anti-inflammatory and anticancer agent [101]. However, due to its low water solubility, THC has only limited pharmacological activities. To overcome this drawback, Plyduang *et al*. [40] conjugated THC with CMC. In addition to being approved by FDA as a common excipient, CMC is inexpensive, has good compatibility to the skin and mucous membranes and presents suitable groups, which allow functionalization. The CMC derivative was conjugated with PABA (*p*-aminobenzoic acid) or PAH (aminohippuric acid)-diamine linkers, both of which can be cleaved by colonic bacteria. This property was chosen as the authors intended to prepare a colon-specific drug delivery material for the treatment of colonic cancer. To decrease side effects, the targeted drug delivery to the colon should not release the drug in the stomach and small intestine. After assessing the *in vitro* release of the conjugates (from 65% to 82% after 48 h), the analysis of cytotoxicity, revealed an increased toxicity in the human colon adenocarcinoma cell line HT-29 compared to the normal colon cell line CRL1790. This specificity, however, seemed to depend on the drug *per se* and not to any targeting ability of the delivery materials. The authors also showed that the incubation of the conjugates with gastric or small intestine homogenate resulted in a minor drug release; however, in the presence of cecal content, drug release had a burst. The authors explained this behavior with the action of colonic bacteria, which could have triggered the cleavage of PABA/PAH linkers. Despite being potentially interesting, these data need to be confirmed in physiological model considering the delivery behavior in living animals.

As mentioned before, the use of polysaccharides can be applied to integrate drug delivery and diagnostic/imaging in one system. These types of systems were, for example, investigated by Wu *et al*. [41]. The authors developed a class of multifunctional hybrid nanomaterials through the *in situ* immobilization of CdSe quantum dots (QDs) into the hydroxypropylcellulose-poly(acrylic acid) (HPC-PAA-CdSe) semi-interpenetrating (semi-IPN) nanogels. The fluorescent CdSe QDs is used for particles visualization; the HPC chains can provide rich-OH groups for sequestering CdSe ions into the gel network and stabilizing the in-situ formed CdSe QDs. Finally, the PAA chains are the pH sensitive portions that, depending on the pH, can induce reversible swelling/shrinking of the particles influencing drug delivery. Nanogels were successfully internalized into the mouse melanoma B16F10 cells as evaluated by fluorescence microscopy, giving no signs of morphological damage to the cells. The addition of the anticancer drug temozolomide (TMZ) into nanogel resulted in a significant reduction in B16F10 cell vitality compared to controls. Notably, nanogels did not alter the potency of TMZ as the drug either complexed in nanogels or in the naked form, had similar activity. TMZ is an oral anticancer agent approved for the treatment of newly diagnosed glioblastoma in combination with radiotherapy. The proposed nanogels may be useful to overcome some of the limitation of TMZ, such as the low solubility, the short half-life, and the considerable toxicity. In particular, TMZ toxicity may be reduced by adding to the nanogels cancer-cell targeting moieties. The developed nanogels are certainly promising for optimized drug delivery, but further *in vivo* studies are necessary to confirm their efficacy.

#### 3.1.8. Other Polysaccharide Based Delivery

Other interesting, but thus far not yet deeply investigated, polysaccharides can be used as drug delivery materials. In this regard, a promising class is represented by sulfated polysaccharides. As most of them are negatively charged, they can bind positively charged molecules via electrostatic interaction. A recent study [80] considered the use of *Halomonas maura*, a halophile bacterial species, for its capability to produce huge amounts of sulfated exopolysaccharides particularly rich in sulfate residues. The numerous sulfate residues render this polysaccharide, named mauran (MR), an immune-modulating and anticancer agent itself. In the study, NPs composed of MR and CS were synthesized and filled with 5-FU (5-FU-MR). MR/CS NPs were synthesized by simple polyelectrolyte complexation of the anionic MR and the cationic CS. The obtained NPs, in the shape of spheres with a smooth surface, released 5-FU mainly via diffusion. No significant toxicity was observed in mouse connective tissue fibroblast cells (L929). Additionally, the authors could show that in MCF7 cells, the anti-proliferative capacity of 5-FU was enhanced following the release from NPs, compared to the naked drug. An explanation given for this observation was the increased 5-FU retention into the cells compared to the naked drug form. This feature together with the capacity to exert a sustained released of the drug (up to 10–12 days), indicate the interest for this kind of NPs, whose characterization should be further deepened to fully understand the effectiveness in drug release *in vivo*.

Another example of a not deeply studied polysaccharide is arabinogalactan (AG), a natural polymer extracted from Larix tree. The group of Pinhassi [81] created a smart system to ameliorate methotrexate (MTX) administration. MTX is a chemotherapeutic agent approved by FDA for the treatment of different cancers (breast cancer, acute lymphocytic leukemia). However, its administration causes different side effects. In the study of Pinhassi *et al.* [81], AG was conjugated with folic acid (FA), to allow the targeting to cancer cells which typically overexpress FA receptor. The drug loaded NPs displayed 6.3-fold increased cytotoxic activity in FR-over-expressing cells (AA8 Chinese hamster ovary cell line) compared to their FR-lacking counterparts. This interesting targeting ability will need to be confirmed in animal models; additionally it would be interesting to test whether the NPs could be conjugated with different targeting moieties to further improve the specificity of action.

### 3.2. Polysaccharides for the Delivery of Nucleic Acid Based Drugs

In addition to the clinically approved drugs, also other experimental molecules with potential therapeutic value can benefit from the polysaccharide-mediated delivery. Among the experimental molecules, we focussed on NABDs. These compounds, constituted by short sequences of either DNA or RNA, includes antisense oligonucleotides, decoys oligonucleotides, aptamers, triple helix forming oligonucleotides, DNAzymes, Ribozymes, small interfering RNAs (siRNAs) and micro interfering RNAs (miRNAs) [102,103,104]. As all NABDs are made of short nucleic acid molecules, they have comparable physical-chemical features. It follows that they display similar requirements in terms of the delivery material. Thus, whereas in this review we will focus on polysaccharide-based delivery systems for siRNAs/miRNAs, the same systems can be in principle effective also for the other class of NABDs.

In the next section, we will present the general biological features of siRNA/miRNA (Section 3.2.1) together with the specific problems related to the release of these molecules (Section 3.2.2). Then, we will present (Section 3.2.3, Section 3.2.4, Section 3.2.5, Section 3.2.6 and Section 3.2.7) the employment of representative and widely studied polysaccharides for NABD delivery.

#### 3.2.1. Small and Micro Interfering RNA Molecules

The RNA interference (RNAi) is a cellular process characterized by the degradation, in a sequence dependent fashion, of a target RNA by short double stranded RNAs (dsRNAs) [105]. miRNAs and siRNAs are the short dsRNAs able to trigger RNAi. While miRNAs are synthesized within the cell, siRNAs are commonly of exogenous origin deriving, for example, from viruses and transposons. With regard to the action mechanism, of the two RNA filaments constituting miRNA/siRNA, one (the so-called antisense strand) is up-taken by the cellular protein complex termed RISC (RNA-induced silencing complex), while the other (called sense strand) is discarded and does not take part to the silencing process. The antisense strand drives RISC to the target RNA that is bound via either a perfect or a partial complementarity in the case of siRNA or miRNA, respectively. In the case of miRNAs, the binding triggers the down-regulation of target gene expression most often by interfering with the translational machinery [106] and, in some cases, by inducing target RNA degradation by RISC. In contrast, siRNAs repress target gene expression only via RISC-mediated target RNA degradation. Due to the short length, miRNAs and siRNA can be easily chemically synthesized and used to target the mRNAs of genes causing disease. In this regard, there are several examples of the potential therapeutic value of miRNAs and siRNAs [107,108,109,110,111,112,113].

#### 3.2.2. The Delivery Problems

**Figure 6:**
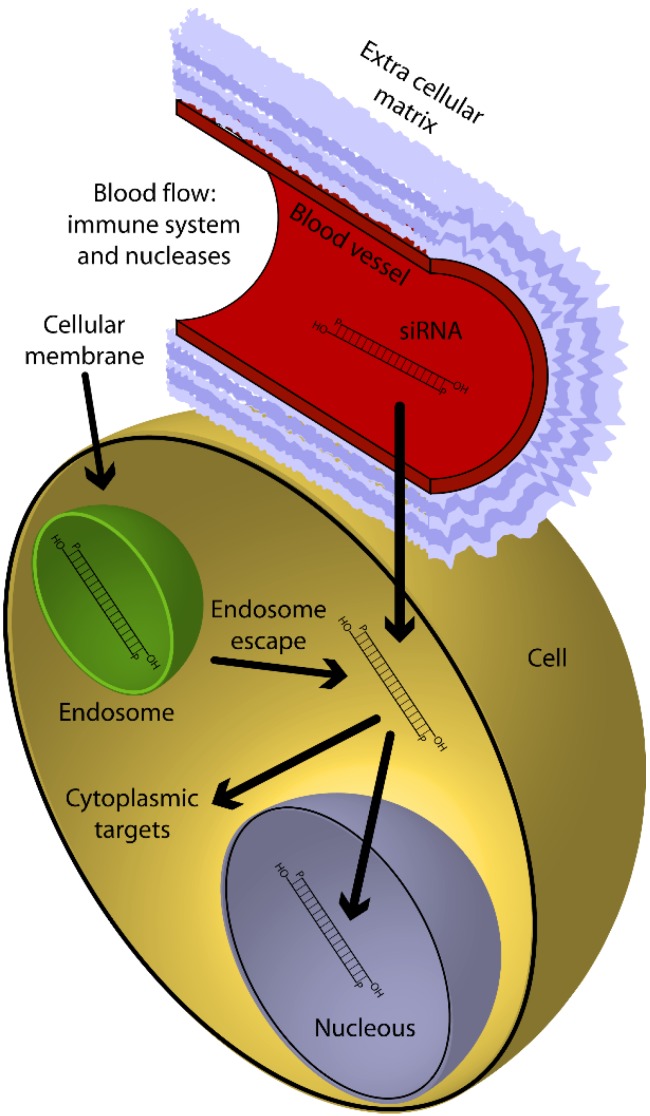
Biological obstacles in NABDs delivery.

Whereas the therapeutic potential of miRNAs/siRNAs, which from now on will be collectively indicated as NABDs, is generally recognized, their practical use is limited. This is substantially due to the absence of optimal delivery systems able to efficiently protect and direct them to the target tissue. If administered systemically (vascular route) in the naked form (Figure 6), NABDs have to face with the obstacle of blood nucleases, which can rapidly trigger their degradation. Additionally, naked NABDs tend to be eliminated by the reticulo-endothelial system, by kidney filtration [114], and, in some cases, tend to activate the innate immunity [115,116]. The crossing of the vascular wall before reaching the diseased tissue, represents another obstacle to NABDs action. The efficiency of this process is strongly influenced by the size of the endothelium fenestration, a feature that can vary considerably among tissues. For example, the liver endothelium is highly fenestrated, thus favoring extravasation [117] and accumulation of NABDs in the liver.

Once in the desired tissue NABDs have to overcome the problem of the cell membrane crossing. This step is unfavorable due to the negatively charged surface of cellular membranes, which tend to repulse the negatively charged phosphate groups present in NABDs. Additionally, the hydrophilic nature of NABDs disfavors the crossing of the hydrophobic layer of the cell membrane. In the cells, NABDs are then susceptible to further degradation by cellular nucleases. In the cell, NABDs are also subjected to the cellular trafficking issue. This last aspect is relevant [118,119] as, following cellular uptake, NABDs can be sequestered into cytoplasmic vesicles named endosomes where they remain entrapped with no possibility to reach the target mRNA into the cell cytoplasm.

Together all the above considerations clearly indicate that naked NABDs have no chance to exploit their therapeutic action. A strategy undertaken to overcome NABDs degradation is based on the introduction of chemical modification into their structure to make them resistant to nucleases-mediated degradation. A second possibility is represented by the complexation/binding to different synthetic materials. While the discussion of the NABDs chemical modification [111] goes beyond the scope of the present work, the strategies adopted to complex NABDs with synthetic materials represents the topic of this review. In particular, among the several materials available [111], we concentrate on the use of polysaccharides as delivery substances due to their wide use and versatility. The reported examples are described below and summarized in Table 3.

**Table 3:** Polysaccharide-based delivery of NABDs.

	System	Structure	Drug	Disease	*In vitro* Model	*In vivo* Model	Ref.
**Chitosan**							
1	Bio-CS-g-PEI	NPs	siRNA	Ovary cancer	HeLa	–	[23]
					OVCAR-3		
2	CS-TAT	NPs	siRNA anti luciferase/survivin	Brest cancer	MCF7	Mouse	[24,25]
	CS-nonaArg						
3	CS-γPGA	NPs	siRNA anti luciferase/GFP	–	HT1080	–	[26,27]
4	psi-Pgp-GCt	NPs	siRNA anti Pgp	Brest cancer	MCF7	Mouse	[28]
**Hyaluronic Acid**							
5	HA-Chol/2b	NPs	siRNA anti RFP	–	B16F10	–	[34]
6	PEG-HA-NP	NPs	siRNA anti Pgp siRNA anti GGCT	Breast cancer	MCF7	Mouse	[35,36]
7	CAP/dopa-HA	NPs	siRNA anti luciferase	–	HT29	Mouse	[37]
8	CAP-HA-DPA/Zn	NPs	siRNA anti luciferase	–	DU145	Mouse	[38]
					143B HCT116		
**Dextran**							
9	Ac-DEX-spermine	NPs	siRNA anti luciferase	–	HeLa	–	[47,48]
10	DexS/pArg	NPs	siRNA anti GFP	–	HeLa	–	[49]
11	DexS/pArg/HY	NPs	siRNA anti SPARC	–	Fibroblast	–	[50]
**Pullulan**							
12	P-PEI-FA	NPs	siRNA anti luciferase	–	HeLa	–	[52]
13	Ps	NPs	siRNA	–	MDA-MB-231	–	[53]
**Other**							
14	CS/alginate	hydrogel	siRNA anti TNFα	Colitis	–	Mouse	[42]
15	CS/alginate	hydrogel	siRNA anti CD98	Colitis	Colon-26 RAW 264.7	Mouse	[43]
16	PEI/alginate	NPs	siRNA anti VEGFR-3	–	EPCs	–	[44]
17	PEG-Tf-CD	NPs	siRNA anti RRM2	–	–	Mouse	[120,121]
						Non-human primate Clinical trial	

#### 3.2.3. Chitosan Based Delivery

CS has been widely used as NABDs delivery agent mainly because it contains positively charged amino groups that permit the electrostatic interactions with the negatively charged NABDs. Darvishi *et al*. [23] have proposed the grafting of polyethyleneimine (PEI) on CS backbone. PEI is a synthetic polymer with a repeating unit of an amine group with two CH_2_ spacers. Due to the presence of protonable amino groups in every third position, PEI has a high cationic density under physiological pH, which allows the spontaneous formation of polyelectrolyte complexes with nucleic acids. In addition to PEI, Darvishi *et al.* [23] added biotin (Bio-CS-g-PEI) whose receptor tends to be over-expressed in cancer cells. Bio-CS-g-PEI resulted to be water-soluble at physiological pH and had optimal transfection efficiency for siRNA at the Bio-CS-g-PEI/siRNA weight ratio of 30. Moreover, Bio-CS-g-PEI/siRNA transfection efficiency was 44% and 22% higher than that of CS-g-PEI/siRNA and PEI/siRNA complexes, respectively, in the HeLa tumor cells. Worth noting is the fact that, in OVCAR-3 cells, a model of human ovary cancer cells over-expressing biotin receptors on the cell membrane, the transfection efficacy was 74% and 23% higher, compared to CS-g-PEI and PEI complexes, respectively. Together, these data strongly support the targeting potential of Bio-CS-g-PEI/siRNA in cancer cells over-expressing biotin receptors. Confirmatory experiments are now necessary in *in vivo* model.

As an additional and interesting strategy to improve the efficiency of CS/siRNA transfection, cells penetrating peptides were used in combination with CS. In two different works it has been demonstrated that the grafting of TAT peptide [24] or nona-arginine peptide [25] ameliorated not only the quantity of internalized siRNA but also the silencing effect of siRNA compared to the siRNA delivered by unmodified CS. More in details, nona-arginine and TAT increased in MCF7 cells the uptake of 2.5 and 13 folds, respectively, compared to the unmodified CS. Notably, both for nona-argine or TAT grafted CS, the cyto-compatibility was definitively superior to unmodified CS. Additionally, the siRNA mediated silencing effect was excellent reaching 75%. TAT-grafted CS also showed its effectiveness *in vivo*, delivering a siRNA targeted against the mRNA of survivin, a protein known to inhibit cell apoptosis, thus favoring tumor cell survival. Following direct intra-tumor injection, TAT-CS-siRNA complexes were able to significantly reducing tumor growth in breast tumor-bearing mice in comparison to naked siRNA and saline treated mice. Interestingly, whereas mice treated by saline or naked siRNA developed lung metastasis, this was not the case for TAT-CS-siRNA treated animals [25], further showing the effectiveness of this delivery material.

Despite being the above reported success in the use of CS as siRNA delivery material very encouraging, it should be kept into consideration the fact that the strong interaction CS-siRNA, may negatively affect the siRNA release into the cell. This seems to be particularly true for high molecular weight (MW) CS [122], even if high MW CS can efficiently protect siRNAs from degradation. On the other hand, low MW CS is not optimal in siRNA protection against degradation in the extracellular environment, but it can efficiently release the siRNA in the cell [123]. Together, these observations indicate that the balancing between CS protection and release properties of siRNAs needs to be optimized [124]. Additionally, this balancing may need to be optimized also in relation to the target cell. For example, CS with intermediate chain lengths mediated an efficient siRNA delivery in H1299 (human lung carcinoma cell line), in the MCF7 and in HUVEC (human umbilical vein endothelial) [125]. However, very poor delivery was observed by us in the human hepatic cancer cell line JHH6 (Farra *et al.*, unpublished results), suggesting a cell-dependent efficiency of uptake.

With the purpose to improve CS-mediated delivery of siRNAs via the reduction of CS attraction towards siRNAs, Liao *et al*. conjugated to CS a negatively-charged poly(γ-glutamic acid), together with a γ-glutamyl unit at its N-terminal end (γPGA) [126] to form CS/γPGA complexes. As γPGA can be recognized by a membrane protein, γ-glutamyl transpeptidase (GGT), highly expressed in most mammalian cells, the entire CS/γPGA complexes were up taken by means of a receptor mediated endocytosis. Moreover, the authors showed that, using a double stranded DNA as a prototype of a nucleic acid molecule, the CS/DNA/γPGA complexes was gradually unpacked into the cell due to the electrostatic repulsion induced by the incorporated γPGA, which facilitated the intracellular release of DNA. This system was also used to deliver two disulfide bond-conjugated and PEGylated siRNAs (PEG, polyethylene glycol) directed against the mRNAs of luciferase and GFP (green fluorescence protein) [26]. To study the effects of the two siRNAs complexed with CS/γPGA (CS/siRNA/γPGA), the HT1080 cell line stably transfected with GFP/luciferase reporter gene plasmid was used. The authors could show that following endosomal escape, the negatively charged γPGA of the CS/siRNA/γPGA was able to disrupt the strength of electrostatic interactions between the conjugated siRNAs and the protonated CS. This facilitated the release of the two siRNAs into the cytosol where the intracellular glutathione could reduce the disulfide bond of the conjugated siRNAs making them free and, thus, able to silence the respective target mRNA (GFP and luciferase). The maximum suppression of gene expressions (80%) was observed at day five after administration and persisted, although progressively reduced in extent, up to day eleven. Notably, whereas at day one CS/siRNA/γPGA could reduce gene expression down to 28%, the CS/siRNA complex only reached 50%, showing the relevance of γPGA [27]. Despite that these data are very interesting, the comparison with a LF mediated delivery could have better defined the efficacy of the system.

**Figure 7:**
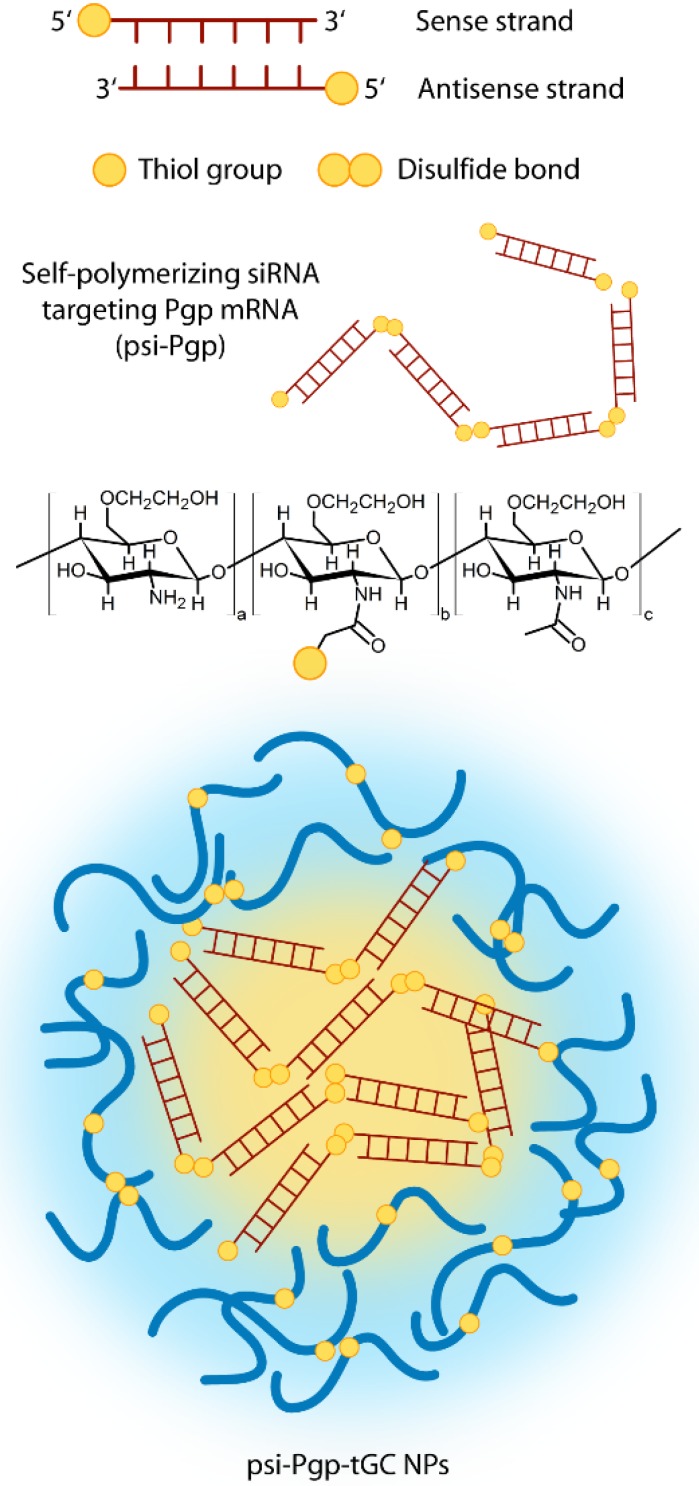
CS-based delivery for siRNAs proposed by Ji Young Yhee *et al.* [28].

An original CS-based delivery for siRNAs is the one proposed by Ji Young Yhee *et al*. [28]. The authors used glycol chitosan (GC) polymers bearing thiol groups (GCt) (Figure 7).

The siRNA of interest was also thiolated at the extremities, thus inducing the formation of a sort of poly-siRNA (psi). GCt and psi were then mixed and allowed to bind by electrostatic interactions and by the chemical conjugation via thiol groups. The siRNA sequence used was directed against the mRNA of P-glycoprotein (Pgp), which mediates the therapeutic drug efflux from cancer cells, thus inducing cancer cell multi drug resistance (MDR). The resulting NPs named psi-Pgp-GCt, were spherical with an average hydrodynamic diameter of approximately 270 nm. The NPs could protect siRNA molecules from the degradation exerted by cellular and extracellular nucleases. At high particle concentrations (59.4 μg/mL, 400 nM of siRNA of irrelevant sequence), almost no reduction in the vitality of MCF7 was detected by MTT assay; in contrast, the siRNA of irrelevant sequence, released from lipofectamime (LF), displayed 50% reduction in cell vitality. Additionally, the psi-Pgp-GCt NPs were able to enter the cytoplasm at neutral to acidic pH (pKa of chitosan= 5–7) and to escape the lysosomes due to the change of endosomal pH. With regard to the functional effect, psi-Pgp-GCt NPs (100 nM of siRNA against Pgp) induced significant Pgp suppression in MCF7 cells which, however, was somewhat inferior to that obtained by the commercial available LF. Despite this, the reduction in Pgp levels reached by the produced NPs, was able to decrease the extrusion from the cells of the anti-cancer drug DOX. Thus, these data suggest that psi-Pgp-GCt may induce changes in drug sensitivity. This was proven by showing the reduced cell vitality following DOX administration in psi-Pgp-GCt transduced compared to non-transduced cells. *In vivo*, the bio-distribution of psi-Pgp-GCt delivered via the tail vein in MCF7 tumor-bearing mice, showed a predominant localization at the tumor site, which was about three times higher than in the control. Moreover, psi-Pgp-GCt co-treatment with DOX significantly enhanced the anti-cancer activity of DOX, as shown by the fact that the mean tumor volume of psi-Pgp-GCt + DOX group was significantly reduced compared to DOX treated animals. Thus, these data confirm, *in vivo*, the induction of drug sensitivity by psi-Pgp-GCt. Notably, the authors could also prove the significant reduction of DOX toxicity in animals treated by psi-Pgp-tGC NPs.

#### 3.2.4. Hyaluronic Acid Based Delivery

The negative charge of HA does not allow an effective binding to the negatively charged NABDs. To overcome this limitation, HA has been used in combination with cationic polymers to form polyelectrolyte complexes by supramolecular self-assembly able to bind NABDs. In this regard, different molecules including PEI, Spermine [127], Poly l-Lysine [128], and other [1] have been tested. A notable example is represented by the work of Choi *et al*. [34]. The authors developed a siRNA delivery system based on HA-containing NPs with the HA backbone conjugated with cholesterol (HA-Chol NPs). HA-Chol NPs where generated with a variable number of cholesterol per 100 sugar residues (from 2.6 to 24). Then, the siRNA was combined with the 2b protein, a virus derived protein able to bind double stranded RNA (dsRNA). The author demonstrated that it was possible to encapsulate the 2b/siRNA complex into the HA-Chol NPs to form the HA-Chol/2b/siRNA complex. This particle showed a loading content of siRNA up to 11% *w*/*w* for NPs containing the higher amount of cholesterol. Using a siRNA directed against the red fluorescence protein (RFP), the authors showed that, in B16F10 cells expressing RFP, HA-Chol/2b/siRNA particles efficiently reduced RFP mRNA and protein levels. Worth noting, the commercially available LF 2000 was significantly less effective than the HA-Chol/2b/siRNA complexes. This elegant delivery system needs now to be tested in more complex scenario *in vivo*, using therapeutic siRNAs to fully demonstrate its delivery power.

HA can be also used as coating for positively charged NPs. The positive charge on the NP can interact with negatively charged serum protein, which can displace siRNA from the NP. This, in turn, results in the reduction of the amount of siRNA delivered to the target cells, and, to a certain extent, also in a possible off-targeting of the free siRNA with unpredictable effects in terms of toxicity. The coating with HA can minimize the siRNA displacement from the NPs thus improving cellular uptake, biocompatibility and colloidal stability. In this regard, an interesting example has recently been reported by Ran *et al*. [35]. The authors covalently linked HA to PEG molecules and used the resulting PEG-HA compound to coat liposome-siRNA NPs. Whereas liposome are commonly used as siRNA delivery molecules, they are not optimal for systemic delivery of siRNA and, thus, need to be conjugated with “smart molecules”. The addition of PEG can efficiently shield the positive charge of liposome, thus reducing the siRNA displacement by negatively charged serum protein, from the NP (PEG-NP). However, PEG tend to reduce the cellular uptake and compromise endosome escape. HA can contribute to overcome these problems, as shown by Ran *et al*. [35] with the generation of liposome NPs coated by PEG-HA (PEG-HA-NP). PEG-HA-NP, with an average diameter of 190 nm and a negative surface charge, were stable in fetal bovine serum up to 24 h. Compared to NP, PEG-HA-NP carrying a siRNA directed against the mRNA of the P-glycoprotein, resulted in a more efficient down regulation of target mRNA in MCF7 cells. Because of the good stability in serum, the systemic administration of PEG-HA-NP could efficiently deliver the siRNA in tumor bearing mice reaching a 34% P-glycoprotein down regulation. In contrast, no activity was detected for siRNA delivered from either NP or HA-NP. As P-glycoprotein is involved in the efflux of drugs outside the cells, in so preventing drug accumulation into the cell its down regulation by the PEG-HA-NP delivered siRNA can contribute to reduce the problem of drug resistance in breast cancer cells. To further confirm the reliability of the PEG-HA-NP approach, in a different work by the same author [36], this system was successfully tested in MCF7 cells for the delivery of another siRNA targeted against γ-glutamylcyclotransferase (GGCT), a protein upregulated in various types of cancer cells. Since HA can selectively bind CD44, the use of PEG-HA-NP may be in principle extended to the targeting of the many different CD44 over-expressing tumor cells. However, the extent of the increased efficacy of PEG-HA-NP in CD44 over-expressing compared to under expressing cells remains unclear since the authors did not specifically address this aspect in their work.

HA has been combined with calcium phosphate (CAP), a natural inorganic material that can be found in bone and teeth. This material has been frequently used as gene delivery agent due to its biocompatibility, biodegradability, and ability to encapsulate negatively charged genetic material via the chelation of calcium ions during the process of calcium-phosphate crystals formation. The lack of delivery specificity and the uncontrollable growth of CAP crystals in a physiological solution has, however, limited its use as gene delivery system *in vivo*. Very recently, the conjugation of CAP with 3,4-dihydroxy-l-phenylalanine (DOPA), an unusual amino acid found in adherent threads of marine mussels, has been proposed as a novel device for the targeted siRNA delivery to tumors [37] in conjunction with HA. HA-DOPA was first synthesized and then used to stabilize the CAP/siRNA NPs, upon absorption on their surfaces. Due to the adhesive properties, the DOPA molecules have the role to bind to the surface of CAP particles while the hydrophilic HA backbone exerts protective functions and serves as a targeting moiety for CD44+ cancer cells. At the weight ratio DOPA-HA/siRNA of 60, the siRNA incorporation efficiency was about 84%; additionally, no significant cytotoxicity was observed as evaluated by MTT test. CAP/DOPA-HA containing an anti-luciferase siRNA (DOPA-HA to siRNA weight ratio of 60), reached in CD44+ human colon carcinoma cells (HT29-luc) over-expressing luciferase, 50% reduction of target gene expression. In addition to showing that the uptake mechanism of CAP/siRNA/ DOPA-HA was regulated by receptor-mediated endocytosis, the authors also demonstrated that the acidic endosome pH promotes CAP dissolution determining an increase in the concentration of calcium and phosphate ions. This, in turn, induces the swelling and disruption of the endosomal membrane due to increased internal osmotic pressure with the consequent siRNA release. In animals bearing HT-29-luc tumor xenografts, CAP/siRNA/DOPA-HA administered via the tail vein, accumulated predominantly in the tumor mass and, in part, also in the liver. This last phenomenon may be attributed to the presence of the HARE receptor in hepatocytes; however, no signs of hepatic toxicity were detected following the histological evaluation of the liver and the evaluation of the liver function tests. In our opinion, this observation needs further careful investigations. Notably, CAP/siRNA/DOPA-HA was able to significantly suppressing the expression of the target gene (luciferase) after a single dose (0.6 mg/kg) administered via the tail vein.

**Figure 8:**
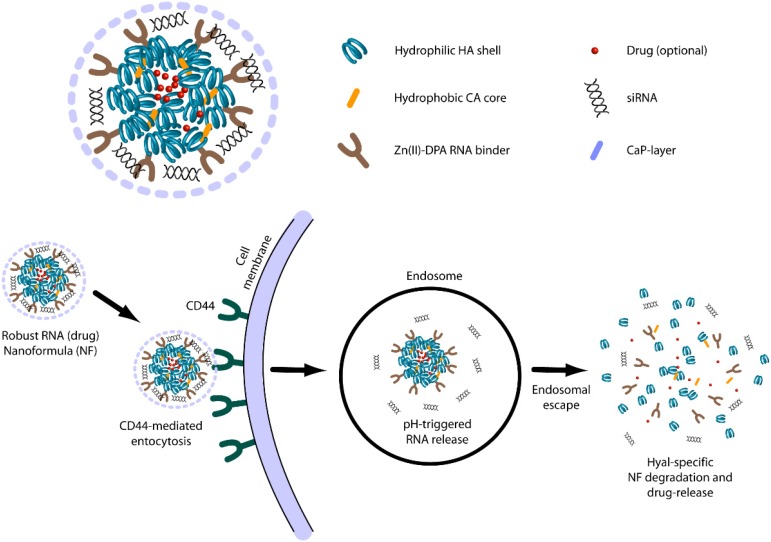
HA-based delivery for siRNAs proposed by Choi *et al.* [38].

Another recent and interesting work based on the use of CAP and HA has been reported by Choi *et al*. [38] (Figure 8). The siRNA NP is composed by four major components: (i) a hydrophilic HA shell; (ii) a hydrophobic 5β-cholanic acid (CA) inner core; (iii) a phosphate receptor Zn(II)-dipicolylamine (DPA/Zn); (iv) a calcium phosphate layer (CAP). The HA shell confers the ability of NPs to bind CD44 positive tumor cells, CA offers the possibility to load into NPs hydrophobic drugs and DPA/Zn allows the binding to siRNA. In particular, DPA/Zn binds siRNAs via specific interactions between coordinated zinc ions of DPA and anionic phosphates of the RNA. Due to the significantly high phosphate concentration in the bloodstream, which can perturb the siRNAs-DPA/Zn binding, the author added CAP layers onto HA-DPA/Zn/RNA by *in situ* mineralization of sequentially added calcium and phosphate ions. In this way, the CAP layer can protect the DPA/Zn/RNA interaction in the biological environment. Additionally, as above discussed, at the low pH of endosome, the CAP is dissolved, favoring the endosome escape of DPA/Zn/RNA. Notably, the phosphate ions released from CAP competitively bind with DPA/Zn receptors triggering the HA-siRNA release. Subsequently, the HA backbone can be degraded by the enzyme hyaluronidase, abundant within tumor cells, eventually resulting in the release of the free siRNA. Worth note is the fact that this kind of NPs can allocate, in addition to siRNAs or other NABDs, also hydrophobic drugs due to the presence of CA. Thus, this NPs can be used to exert synergistic therapies mediated by siRNAs and hydrophobic drugs, such as those commonly used in cancer chemotherapy. Using red fluorescent labeled siRNA (Cy3-siRNA) Choi *et al*. [38] could detect in the prostate tumor cells DU145 and 143B, a strong fluorescence signal, which was about three times more intense than that obtained delivering the siRNA by liposomes. Moreover, releasing a siRNA directed against the luciferase gene in 143B tumor cell line over-expressing luciferase, the authors observed thirty times more activity of the NP-siRNA complexes compared to the liposome-mediated delivery of siRNA. In a HCT116 xenograft mouse model, NPs intravenously injected were able to dramatically reduce the expression of the target gene (Luciferase) over-expressed by the HCT116 tumor cell line. Interestingly, it was also demonstrated the feasibility of miRNA delivery by the prepared NPs. Finally, the authors also provided evidences of the possibility to co-deliver the anticancer drug PTX and siRNA to the HCT116 cancer cells. Notably, in an evolution of the above NPs, the authors [129] showed the feasibility to convert the functional group of Zn-DPA into other groups including a carboxylic or thiol group; moreover, the DPA analog can be covalently attached to different formulations suitable to generate multifunctional delivery materials via standard bio-conjugation techniques. Together, these data fully underline the great versatility of the proposed system.

#### 3.2.5. Dextran Based Delivery

Thus far, several types of dextran-based NPs have been generated for NABD delivery. From the first attempt where dextran alone was bound via electrostatic interaction to NABDs, more complex solutions have been proposed. For example, Naeye *et al*. decorated the dextran/NABDs NPs with a hydrophilic PEG shell for the intravenous delivery route [130,131]. Whereas PEGylation did not prevent the partial dissociation of NABDs in human plasma, it was able to prevent NPs aggregation and the lysis of red blood cell. However, a clear charge dependent interaction with platelets and leukocytes was observed. On one hand, these results suggest the positive NPs features able to prevent a significant interaction with red blood cells; on the other hand, they indicate that further improvements aimed at the reduction of platelet interaction are necessary.

Another example of dextran NPs modification consisted in the conjugation of an acetal-modified dextran (Ac-DEX) with spermine as NABD delivery material [47]. The cationic nature of spermine improved the NPs complexation with the negatively charged siRNA and facilitated the cell membrane binding. Additionally, the Ac-DEX-spermine NPs resulted to be efficiently released from endosome, thus allowing the siRNA distribution with the cell cytoplasm [48]. This in turn resulted in an efficient silencing of the expression of the luciferase gene in HeLa cells expressing luciferase.

Very recently, dextran was used to encapsulate NPs containing NABD and PEI [49]. In particular, the authors encapsulated NABD/PEI with multiple layers of dextran sulfate (DexS) and poly-l-arginine (pArg). The idea below this approach is that the DexS/pArg layers can protect the NABD in the extracellular environment and prevent the known toxic effects of PEI. Once in the cellular environment, the DexS/pArg layers are progressively degraded, allowing PEI to mediate the known endosomal escape effect, thus favoring NABD translocation to the cytosol. As a cellular model it was used the HeLa cell line expressing the GFP against which the effect of an anti-GFP siRNA was tested. DexS/pArg capsule increased siRNA activity by about two to three times compared to the non-encapsulated PEI/siRNA. Notably, it was also shown that the minimal concentration of siRNA able to exert a visible effect was of 2.6 nM; in contrast, for the commonly available transfection reagents a concentration of about 100 nM was necessary. This data strongly support the potency of the system proposed that, in addition, displayed negligible cytotoxicity.

In a similar approach to that above reported, DexS and pArg were recently used to coat a core of hydroxyapatite (HY) particles [50]. The technique used is the layer-by-layer (LbL) self-assembly, a method based on the electrostatic interactions between positively (pArg) and negatively (DexS) charged polymers. A siRNA was inserted within the two most external pArg layers with an efficiency of about 97%. The resulting NPs yielded a size of 350 nm with a superficial charge of +43 mV. Initial studies performed using a fluorescently labeled siRNA indicated an efficient uptake into fibroblast with a clear cytoplasmic but not nuclear localization. The nuclear exclusion was explained with the size of the NPs, which was probably too big (350 nm) to enter the nucleus. NPs displayed a good endosome escape and a subsequent defoliation of the external pArg layers that allowed the siRNA homogeneous release into the cytoplasm. For the functional studies, a siRNA target against the secreted protein, acidic and rich in cysteine (SPARC) was chosen. SPARC is a calcium-binding matricellular protein that modulates cell/extracellular matrix and is involved in the generation of pathological tissue scarring and fibrosis. Thus, SPARC targeting may be a useful approach to modulate pathological fibrosis. The authors could show a significant reduction in the protein and mRNA levels of SPARC in fibroblasts; however, as it was not introduced a control containing a scrambled siRNA embedded into NPs, it is hard to evaluate the real effectiveness of the proposed system.

#### 3.2.6. Pullulan Based Delivery

Due to its non-ionic nature, P functionalization with positive groups is necessary to allow the complexation with NABDs. P use as NABD delivery material has been recently undertaken by Wang *et al*. [52]. In this case, a low MW branched PEI (1000 Da) was grafted onto the backbone of P with succinic acid as a spacer; then PEI was conjugated with a peptide (FA) able to bind FR to generate the complex P-PEI-FA. FR is a tumor marker over-expressed on the cellular membrane of different malignant cells. P-PEI-FA showed significantly reduced toxicity compared to PEI in the HeLa cell line. P-PEI-FA complexation with nucleic acid molecules confirmed the modest toxicity in different cell tumor lines (Human hepatocellular carcinoma HepG2, HeLa and MCF7). The only exception occurred for the African green monkey kidney cell (COS-7) was, at the highest weight ratio tested, 50% reduction of cell viability was observed, thus suggesting a species-specific toxicity. In the HeLa cell line expressing the luciferase gene, P-PEI-FA complexed with an anti-luciferase siRNA, reduced luciferase expression by 82%, showing slightly higher gene silencing efficiency compared to P-PEI-siRNA (71%) and LF 2000-siRNA (76%). Notably, P-PEI-FA displayed also a targeting ability as it was highly effective in transducing the GFP expressing plasmid into the FR expressing HeLa cells but not in the FR non-expressing HepG2 cells. Together, these data suggest the feasibility of the use of P as NABD delivery material; tests in *in vivo* models will better define the efficacy of the proposed system.

In another recent work, it has been investigated the effects of serum on the transduction capability of NPs containing P chemically modified by spermine (pullulan-spermine, Ps) [53]. Serum is known to affect the transduction efficacy as it tends to be adsorbed by cationic molecules thus inducing the displacement of NABDs from NPs. Zhang *et al.* improved our knowledge about the effects of serum demonstrating that serum can significantly influence the NPs size and, thus, the transfection efficacy. In particular, the authors showed that low serum concentration (1.25% and 2.5%) in culture medium results in large particles of Ps-siRNA, while high serum concentration (10% and 40%) leads to small particles of Ps-siRNA. Compared to the small particles, large particles contain more amines eliciting a stronger proton sponge effect, which determines a more effective lysosomal escape of siRNA, and, thus, an improved siRNA biological effect. Notably, the large particles at the serum concentration of 1.25% display a transfection efficacy about seven times higher than that of the commercially available LF in the cancer cell line MDA-MB-231. Despite the higher transfection rate, large particles are more toxic than small ones. These interesting results prove the usefulness of P as NABDs delivery material and indicate the necessity to optimize NABDs also release in relation to serum-dependent effects.

#### 3.2.7. Other Polysaccharide Based Delivery

Whereas the above-described polysaccharides have been often used as NABD delivery materials, other polysaccharides exist with a less frequent use. Among these, an interesting polysaccharide is represented by alginate. Due to its ability to form hydrogels, alginate has been mostly used for the controlled release of small molecule and protein. In this regard, a recent example is represented by the work of Laroui *et al*. [42]. Fab'-bearing TNFα-siRNA-loaded NPs were encapsulated into a matrix of CS/alginate and administered orally to mouse with induced colitis. Being colitis an inflammatory disease, the targeting of a pro-inflammatory cytokine, such as TNF-α, is considered beneficial to relieve inflammation. Moreover, the addition on the NPs of the Fab' portion of the F4/80 antibody, allows the targeting of macrophages, inflammatory cells sustaining colitis. The administration into the mouse colon of TNFα-siRNA-loaded NPs covered by Fab' was significantly more effective than the uncovered NPs in lessening colitis as shown by the attenuation of all parameters of colonic inflammation. Moreover, grafting Fab' onto the NPs improved the kinetics of endocytosis, as well as the macrophage-targeting ability. The use of alginates as releasing hydrogel was also considered in a similar recent work [43]. NPs were functionalized with molecules able to target CD98, an antigen whose over-expression on the surface of colonic epithelial cells and macrophages promotes the development and progression of colitis. NPs, loaded with an anti CD98 siRNA, were tested *in vitro* and *in vivo* embedded into CS and alginate hydrogel. *In vitro*, the NPs significantly reduced the levels of CD98 in the cell lines Colon-26 cells and RAW 264.7 macrophages, along with the reduction of inflammatory cytokines (TNFα, interleukin-6, and interleukin-12). Notably, administered orally to mouse with induced colitis, NPs also significantly reduced the severity of colitis. Collectively, these results show the relevant role of alginates hydrogel in delivering NABD loaded NPs.

Whereas alginates have been mainly considered as delivery hydrogel, only a limited number of works have used this molecule to prepare nanostructure for NABD release. In the past, alginate has been employed to reduce PEI cytotoxicity and Patnaik *et al*. showed successful siRNA transfection in various cell lines *in vitro* with PEI-alginate NPs containing PEI of different MW (25 kDa or 750 kDa) [132]. Very recently, NPs containing 25 kDa branched PEI and alginate were prepared and loaded with a siRNA targeting the mRNA of VEGFR-3 (vascular endothelial growth factor receptor-3) [44]. The VEGF-C/VEGFR-3 signaling pathway is involved in endothelial progenitor cells (EPCs) differentiation towards lymphoangiogenic cells; moreover, lymphangiogenesis is implicated in lymphatic metastasis of tumor cells. Thus, targeting VEGF-C/VEGFR-3 may be a useful approach to down regulate tumor lymphangiogenesis and lymphatic metastasis. NPs, with an average size and surface charge of 139.1 nm and 7.56 mV, respectively, were effective in reducing VEGFR-3 in purified EPCs. A limitation of these data is represented by the fact that only a semi-quantitative test was used to reveal the mRNA level of the siRNA target. Despite this fact, the authors could show the reduction in EPCs differentiation into lymphatic endothelial cells, together with a reduced attitude to proliferate and migrate. Confirmation in *in vivo* model is now necessary to deeply understanding the power of these alginate-based NPs.

Finally, among other polysaccharide able to release NABD, cyclodextrin has to be mentioned. CDs are naturally occurring cyclic oligosaccharides, consisting of α-1,4 linked d-glucopyranose units. They are characterized by an amphiphilic topology as they form structures hydrophilic on the exterior part and hydrophobic in the inner part. This configuration allows CD to carry hydrophobic molecules inside and outside hydrophilic compounds. CD are also indicated as drug delivery materials as they improve cellular delivery, resistance to endonucleases and can reduce immunogenicity. In NABD delivery, CD can be used as either backbone polymer or as derivative grafted on other polymers. Whereas a detailed description of CD as NABD delivery has been given recently elsewhere [1], here we will just concentrate on some notable examples. With regard to the use of CD as backbone polymer, it should be mentioned the works of Davis’ group [133,134,135]. In this case, PEG and transferrin (Tf) were linked to the CD backbone. Tf was introduced to allow the targeting of cancer cells, which often overexpress the Tf receptor. The resulting PEG-Tf modified CD complexes, loaded with siRNA targeting the ribonucleotide reductase subunit M2 (RRM2), showed significant tumor growth inhibition in murine models [120]. Moreover, dose-escalating studies in non-human primates demonstrated that PEG-Tf modified CD complexes were well tolerated. This delivery approach named RONDEL™ (“RNAi/Oligonucleotide Nanoparticle Delivery”, Calando Pharmaceuticals) was tested in the first in-human phase I clinical trial administered to patients with solid cancer refractory to standard therapies [121]. NPs were detected in post-treatment tumor biopsy sections together with a significant down-regulation of RRM2.

With regard to the use of CD as derivative molecule grafted on other polymers, we believe the works of Kulkarni *et al*. [136,137] represent relevant examples. CD was used as derivatization molecule for a neutral polymer, *i.e.*, poly(vinyl alcohol) (PVA) polymer bearing PEG and cholesteryl (Chol) or adamantane (Ad) modified grafts. The resulting CD-Chol/Ad-PVA-PEG NPs were able to form complexes with siRNA via electrostatic interactions. Worth note, the siRNA delivered by this NPs had comparable efficacy as the same siRNA released from PEI or LF 2000, while being far less toxic.

## 4. Conclusions

A significant limitation in the administration of conventional anticancer drugs is related to their high toxicity mostly dependent on the impossibility to discriminate between cancer and normal cells. Toxicity may also depend on the presence, in the pharmaceutical preparations, of molecules necessary to improve the poor solubility in water of many anticancer drugs. With regard to NABDs, an emerging class of drug with potential therapeutic value, despite evidence of their effectiveness, the practical use is limited. This mainly depends on the lack of materials able to overcome the delivery problems such as the fast degradation in biological fluids, the difficulties to cross cell membrane and the sequestration into cellular lysosomes. Thus, both for conventional anti-cancer drugs and NABDs, the development of optimal delivery materials may radically improve their therapeutic effectiveness. Additionally, appropriate delivery materials may allow the combined administration of conventional drugs and NABDs, possibly potentiating their therapeutic effects.

Among the several delivery materials studied thus far, polysaccharides represent very attractive molecules as they can be obtained in a reproducible way from natural sources, can undergo a wide range of chemical and enzymatic reactions, are biocompatible, biodegradable and have low immunogenic features. Together, these characteristics make polysaccharides excellent candidates for the realization of “smart” delivery systems capable to release, at the appropriate time and site of action the entrapped drugs. However, the development of such delivery systems is not an easy task due to the several aspects to be considered. First, it is important to optimize the polysaccharide-drug interaction, a variable that depends on the physical–chemical properties of the polysaccharide and of the drug. Second, it is necessary to optimize pharmacokinetics and pharmacodynamics through a careful evaluation of the ADME phenomenon. Third, it is necessary to confer a targeting ability to the polysaccharide carrier to limit the off targeting to healthy tissue. Finally, for the applications considering drug delivery from a gel, it is important to study the release mechanisms of the drug from the gels. Together, these considerations clearly indicate that only a multidisciplinary approach can successfully afford the task of the selection of the optimal delivery materials for the drug and application of interest. Whereas an ideal delivery system is not yet available, the examples reported in the present review indicate that many interesting options, based on the use of polysaccharide, are emerging. Thus, whereas additional research is required, the promising results obtained thus far fully justify further efforts in terms of both economic support and investigations in the field.

## Abbreviations

ATP binding cassette transporter B1	ABCB1
Absorption, Distribution, Metabolism and Elimination	ADME
Arabinogalactan	AG
Biotin	Bio
Calcium phosphate	CAP
Cluster determinant 44	CD44
Cisplatin	CDDP
Cyclodextrins	CDs
Carboxymethylcellulose	CMC
Irinotecan	CPT-11
Chitosan	CS
Daunorubicin	DNR
Doxorubicin	DOX
Double stranded RNAs	dsRNAs
Docetaxel	DTX
Extracellular matrix	ECM
Endothelial progenitor cells	EPCs
Enhanced permeability and retention effect	EPR
Folic acid	FA
5-fluorouracil	5-FU
Flutamide	FLT
Folic acid receptor	FR
Förster resonance energy transfer	FRET
Glycosaminoglycans	GAGs
Green fluorescence protein	GFP
γ-glutamylcyclotransferase	GGCT
Hyaluronic acid	HA
HA receptor for endocytosis	HARE
Hydroxypropyl methylcellulose	HPC
Hydroxyapatite	HY
Interpenetrated polymeric networks	IPN
Lipofectamine	LF
Low molecular weight chitosan	LMWC
Lymphatic vessel endothelial hyaluronan receptor-1	LYVE-1
Multi drug resistance	MDR
micro interfering RNAs	miRNAs
Mesoporous silica	MPS
Microparticles	MPs
Mauran	MR
Methotrexate	MTX
Molecular weight	MW
Nucleic acid based drugs	NABDs
Nanoparticles	NPs
Pullulan	P
Pullulan-spermine	Ps
*p*-aminobenzoic acid	PABA
Aminohippuric acid-diamine	PAH
Pharmacodynamics	PD
Photodynamic therapy	PDT
Polyethylene glycol	PEG
Polyethyleneimine	PEI
P-glycoprotein	Pgp
Pharmacokinetics	PK
Paclitaxel	PTX
Red fluorescence protein	RFP
Receptor for hyaluronate-mediated motility	RHAMM
RNA-induced silencing complexes	RISC
RNA interference	RNAi
Ribonucleotide reductase subunit M2	RRM2
small interfering RNAs	siRNAs
Acidic and rich in cysteine	SPARC
Transferrin	Tf
Tetrahydrocurcumin	THC
Temozolomide	TMZ
Vascular endothelial growth factor receptor-3	VEGFR-3

## Cell lines

brain glioblastoma (human)	U87MG
breast cancer cell line (human)	MDA-MB-468NL
breast cancer cell line (human)	MDA-MB-231
breast cancer cells (human)	MCF7
cervical cancer cell line (human)	HeLa
colon adenocarcinoma (human)	HT29
colon cancer (human)	DLD1
colon carcinoma cells (human)	HCT116
colon cell line (human)	CRL1790
colon cells (human)	Colon-26
colorectal adenocarcinoma cells (human)	Caco-2
connective tissue fibroblast cells (mouse)	L929
fibroblast (murine)	NIH3T3
fibrosarcoma cell line (human)	HT1080
head and neck squamous cell carcinoma (human)	SCC7
hepatic cancer cell line (human)	JHH6
hepatocellular carcinoma cell (human)	HepG2
kidney cell (African green monkey)	COS-7
lung adenocarcinoma epithelial cell line (human)	A549
lung carcinoma cell line (human)	H1299
macrophage cells (murine)	RAW264.7
melanoma cell line (mouse)	B16F10
non-small-cell lung carcinoma (human)	NSCLC-NCIH358
ovary cancer cells (human)	OVCAR-3
ovary cell line (chinese hamster)	AA8
prostate tumor cells (human)	DU145
prostate tumor cells (human)	143B
umbilical vein endothelial (human)	HUVEC
